# Comparison of gene expression profiles between human erythroid cells derived from fetal liver and adult peripheral blood

**DOI:** 10.7717/peerj.5527

**Published:** 2018-08-31

**Authors:** Amornrat Tangprasittipap, Pavita Kaewprommal, Orapan Sripichai, Nuankanya Sathirapongsasuti, Chonthicha Satirapod, Philip J. Shaw, Jittima Piriyapongsa, Suradej Hongeng

**Affiliations:** 1 Faculty of Medicine Ramathibodi Hospital, Mahidol University, Bangkok, Thailand; 2 Biostatistics and Bioinformatics Laboratory, Genome Technology Research Unit, National Center for Genetic Engineering and Biotechnology, Pathum Thani, Thailand; 3 Thalassemia Research Center, Institute of Molecular Biosciences, Mahidol University, Nakhon Pathom, Thailand; 4 Protein-Ligand Engineering and Molecular Biology Laboratory, Medical Molecular Biology Research Unit, National Center for Genetic Engineering and Biotechnology, Pathum Thani, Thailand

**Keywords:** Fetal liver, Adult peripheral blood, Erythropoiesis, Microarray, Erythroblast, Differential expression

## Abstract

**Background:**

A key event in human development is the establishment of erythropoietic progenitors in the bone marrow, which is accompanied by a fetal-to-adult switch in hemoglobin expression. Understanding of this event could lead to medical application, notably treatment of sickle cell disease and *β*-thalassemia. The changes in gene expression of erythropoietic progenitor cells as they migrate from the fetal liver and colonize the bone marrow are still rather poorly understood, as primary fetal liver (FL) tissues are difficult to obtain.

**Methods:**

We obtained human FL tissue and adult peripheral blood (AB) samples from Thai subjects. Primary CD34^+^ cells were cultured *in vitro* in a fetal bovine serum-based culture medium. After 8 days of culture, erythroid cell populations were isolated by flow cytometry. Gene expression in the FL- and AB-derived cells was studied by Affymetrix microarray and reverse-transcription quantitative PCR. The microarray data were combined with that from a previous study of human FL and AB erythroid development, and meta-analysis was performed on the combined dataset.

**Results:**

FL erythroid cells showed enhanced proliferation and elevated fetal hemoglobin relative to AB cells. A total of 1,391 fetal up-regulated and 329 adult up-regulated genes were identified from microarray data generated in this study. Five hundred ninety-nine fetal up-regulated and 284 adult up-regulated genes with reproducible patterns between this and a previous study were identified by meta-analysis of the combined dataset, which constitute a core set of genes differentially expressed between FL and AB erythroid cells. In addition to these core genes, 826 and 48 novel genes were identified only from data generated in this study to be FL up- and AB up-regulated, respectively. The *in vivo* relevance for some of these novel genes was demonstrated by pathway analysis, which showed novel genes functioning in pathways known to be important in proliferation and erythropoiesis, including the mitogen-activated protein kinase (MAPK) and the phosphatidyl inositol 3 kinase (PI3K)-Akt pathways.

**Discussion:**

The genes with upregulated expression in FL cells, which include many novel genes identified from data generated in this study, suggest that cellular proliferation pathways are more active in the fetal stage. Erythroid progenitor cells may thus undergo a reprogramming during ontogenesis in which proliferation is modulated by changes in expression of key regulators, primarily MYC, and others including insulin-like growth factor 2 mRNA-binding protein 3 (IGF2BP3), neuropilin and tolloid-like 2 (NETO2), branched chain amino acid transaminase 1 (BCAT1), tenascin XB (TNXB) and proto-oncogene, AP-1 transcription factor subunit (JUND). This reprogramming may thus be necessary for acquisition of the adult identity and switching of hemoglobin expression.

## Introduction

Human erythrocytes are produced continuously by proliferation and differentiation of multipotent hematopoietic stem cells (HSCs) in the process of erythropoiesis. During the course of human ontogenesis, the location of HSCs changes, starting from the embryonic yolk sac in which primitive erythrocytes are made. Later in development, HSCs migrate to the fetal liver, where they differentiate to definitive (enucleated) erythrocytes. Fetal liver HSCs then migrate and colonize the bone marrow, which is the site for production of definitive erythrocytes for the rest of life (Dzierzak & Philipsen, 2013). Definitive erythropoiesis is controlled by hormones such as erythropoietin (EPO) and stem cell factor (SCF) which regulate signal transduction pathways. These signalling pathways orchestrate gene expression programs in erythroid progenitor cells involving transcription factors, DNA-binding proteins, chromatin modifiers and noncoding regulatory RNAs (Hattangadi et al., 2011). Although definitive erythropoiesis is similar between fetal and adult stages, fetal liver HSCs have a greater proliferative capacity and are more sensitive to EPO signalling. It is not known whether these differences are intrinsic or are due to the response of HSCs to the different *in situ* microenvironments where erythropoiesis takes place (McGrath & Palis, 2008).

The most distinctive marker of erythroid cells during each developmental stage is hemoglobin (Hb) expression. The hemoglobin molecule is a tetramer composed of two subunits each of *α*-like and *β*-like globin peptides, along with heme moieties necessary for this molecule’s oxygen-carrying capacity. Primitive erythroid cells in the embryo express embryonic hemoglobin comprised of two ε-globin (*β*-like globin) and two *ζ*-globin (*α*-like globin) subunits, which are progressively lost during fetal development as primitive cells are replaced by definitive erythroid cells (Sankaran, Xu & Orkin, 2010). In the fetal stage, the predominant *β*-like globin molecule produced is *γ*-globin (Sankaran, Xu & Orkin, 2010). The *γ*-globin chains combine with adult *α*-globin chains into a stable tetramer forming fetal hemoglobin (HbF, *α*2*γ*2). Shortly after birth, adult hemoglobin (HbA, *α*2*β*2) replaces HbF to become the predominant hemoglobin. This change is mediated in definitive erythroid progenitors by a transcriptional switch from  *γ*- to  *β*-globin. The transition of fetal to adult globin gene expression is known as “hemoglobin switching” during human ontogenesis. This process has been studied intensively because persistent, or reactivated expression of HbF can significantly ameliorate the clinical symptoms of *β*-hemoglobinopathies such as *β*-thalassemia and sickle cell disease.

Although the molecular events responsible for the transcriptional switch from *γ*- to *β*-globin are known (Sankaran, Xu & Orkin, 2010), understanding of definitive erythropoiesis, and in particular how this process changes overall from fetal and adult stages, is far from complete. Understanding of erythropoiesis requires detailed study of the transcriptomic changes that underpin this process. Comprehensive gene expression patterns during human erythropoiesis in the adult stage have been described in several studies (Keller et al., 2006; Li et al., 2012; Merryweather-Clarke et al., 2011; Peller et al., 2009; Singleton et al., 2008; Sripichai et al., 2009; Xu et al., 2012). In these studies, erythroblasts were obtained from *in vitro* culture of peripheral blood samples with differentiation controlled by external factors added to the growth medium. The knowledge gained from these studies was incrementally driven by improvements in isolating highly pure cultured erythroblasts at different stages of adult erythropoiesis. In the study by Merryweather-Clarke et al., populations of cultured erythroblasts were isolated using flow cytometric cell sorting, which revealed new patterns of gene expression not seen previously (Merryweather-Clarke et al., 2011).

The most intriguing question of how erythropoiesis differs between fetal and adult stages is difficult to address adequately as it requires study of primary fetal liver tissue, which is difficult to obtain. Human embryonic stem cells or induced pluripotent stem cells have been used as surrogates for obtaining fetal liver-like erythroid cells; however, the high expression of embryonic ε-globin in these cells means that they may not accurately reflect erythroid cells from primary fetal liver (Chang, Bonig & Papayannopoulou, 2011). To our knowledge, only two studies have explored transcriptomic profiles of erythroid cells derived from primary human fetal liver cells using microarray (Xu et al., 2012) and RNA-seq (Yang et al., 2013) platforms. The study by Xu et al. (2012) induced differentiation of primary fetal liver cells in culture for up to 7 days in a two-phase serum-free medium, in which erythroblast differentiation was induced by adding hormones in the second phase after expansion. The study by Yang et al. (2013) cultured primary cells for 14 days in an alternative single-phase medium in which hormones for differentiation were supplied throughout the culture period.

Although common genes with differential expression between fetal and adult stages were found between the Xu et al. (2012) and Yang et al. (2013) studies, methodological differences confound comparison of gene expression patterns. Moreover, small sample sizes in both studies limit power to detect all genes with differential expression. The Xu et al. study obtained two fetal and three adult stage samples, whereas the Yang et al. study described expression profiles from embryonic to adult stages with a single replicate of each stage. We conducted a new microarray study comparing gene expression profiles of basophilic erythroblasts (CD71^high^/CD235a^+^) derived by *in vitro* culturing from five fetal liver (FL) and five adult peripheral blood (AB) CD34^+^ cells using Affymetrix GeneChip^®^ Human Gene 2.0 ST Arrays. Meta-analysis combining our data with that from previous study identified the core genes with reproducible patterns of expression between FL and AB erythroid cells.

## Materials & Methods

### Isolation of primary fetal and adult CD34^+^ cells

Human tissue samples were obtained from donors with written informed consent for research use in accordance with the Declaration of Helsinki and approved by Committee on human rights related to research involving human subjects (Faculty of Medicine Ramathibodi Hospital, Mahidol University, Bangkok, Thailand (IRB no. MURA2013/363)). Adult and fetal CD34^+^ cells were isolated from adult healthy donor peripheral blood and fetal liver, respectively. Fetal livers were surgically obtained from five fetuses therapeutically aborted after 13–21 weeks of amenorrhoea. Fetal liver tissue was dissected within 12 hours after collection. CD34^+^ cells derived from fetal liver were prepared using a procedure modified from Bility et al. (2012). Briefly, fetal liver was manually minced into small pieces and homogenized by automated tissue dissociation (gentleMACS Dissociator, Miltenyi Biotech Inc., Auburn, CA, USA) in digestion buffer containing DNaseI grade II, from bovine pancreas (Sigma-Aldrich, St. Louis, MO, USA) and collagenase type I (GIBCO, Grand Island, NY, USA). A single-cell suspension was obtained using a 70 µm cell strainer. Mononuclear cells were isolated from the fetal liver cell suspension and adult healthy peripheral blood by centrifugation on a density gradient of 1.077 g/mL (Lymphoprep, Axis-Shield PoC AS, Oslo, Norway) and subsequently selected for CD34^+^ cells using the positive immune-magnetic selection method (CD34 MicroBead Kit, Miltenyi Biotech Inc.) according to the manufacturer’s recommendations.

### Erythroid differentiation of CD34^+^ cells

Primary CD34^+^ cells were cultured by a two-phase liquid culture system. Cells were cultured for 4 days in phase I medium consisting of Iscove’s Modified Dulbecco’s Medium (IMDM; GIBCO, Grand Island, NY, USA) supplemented with 20% of fetal bovine serum (FBS; Sigma-Aldrich), 300 ng/mL holo-transferrin (holo-TF; PromoCell, Heidelberg, Germany), 25 ng/mL interleukin-3 (IL-3; Cell Signaling Technologies, Beverly, MA, USA), 50 ng/mL human stem cell factor (SCF; Cell Signaling Technologies), and 2 units/mL human recombinant erythropoietin (EPO; CILAG GmbH, Zug, Switzerland). After 4 days, non-adherent cells were collected and reseeded in phase II medium consisting of IMDM supplemented with 20% FBS, 300 ng/mL holo-TF, and 5 units/mL EPO. The culture was maintained under an atmosphere of 5% CO_2_ at 37 °C.

Erythroblast differentiation was monitored throughout culture by flow cytometry analysis using a FACSCalibur flow cytometer (BD Biosciences, San Jose, CA, USA), in which cells were immuno-stained with phycoerythrin-conjugated anti-transferrin receptor (CD71-PE; BD Biosciences Pharmingen, San Diego, CA, USA) and allophycocyanin-conjugated anti-glycophorin A (GPA-APC; BioLegend, San Diego, CA, USA) antibodies. In addition, cell maturation was analyzed by light microscopy of May-Grünwald-Giemsa-stained cytospin preparations. To perform RNA analysis, the culture was sampled at day 8 (4 d in phase I and 4 d in phase II). The culture was continued in phase II medium for another 6 d. At the end of the culturing period at day 14 (4 d in phase I and 10 d in phase II), cells were harvested for analysis of hemoglobin content by high performance liquid chromatography according to the manufacturer’s recommendations (HPLC, VARIANT II hemoglobin testing system; Bio-Rad Laboratories, Hercules, CA, USA).

### RNA isolation and microarray hybridization

Cells harvested at day 8 of culture were stained for CD71 and GPA cell surface markers. Erythroid populations comprising cells with high CD71 and GPA expression were FACS sorted using a BD FACSAria™ III cell sorter (BD Biosciences). Additionally, cells were sorted to remove non-viable cells and debris using forward scatter/side scatter gating. Total RNA was isolated using the RNeasy Plus Micro kit (Qiagen, Mountain View, CA, USA). RNA integrity was determined with the Agilent 2100 Bioanalyzer (Agilent Technologies, Palo Alto, CA, USA); the RNA integrity number for all samples was greater than 8.5. Biotinylated, fragmented cRNA was synthesized from 100 ng of each total RNA sample using the Affymetrix GeneChip^®^ WT PLUS Reagent kit (Affymetrix, Santa Clara, CA, USA), and hybridized to Affymetrix Human Gene 2.0 ST arrays (HuGene-2_0-st, Affymetrix) following the manufacturer’s protocol. Arrays were then washed and stained using the FS450_0002 fluidics protocol and scanned using an Affymetrix 3000 7G scanner. The scanned images were inspected for hybridization efficiency and CEL files generated from AGCC (GeneChip Command Console Software) were imported into Expression Console (EC) 1.3 software for reporting array quality control metrics, including perfect match mean (PM_Mean), background mean (Bgd_Mean), positive and negative probes (POS vs NEG AUC), bacterial spike controls and Poly-A controls.

### Reverse-transcription quantitative PCR (RT-qPCR)

Total RNA samples as described above for microarray experiments were tested by RT-qPCR. First-strand cDNA was synthesized from 1 µg of RNA sample using SuperScript III reverse transcriptase (Invitrogen) following treatment with 1 unit of DNase I (Thermo Fisher Scientific Baltics UAB, Vilnius, Lithuania) according to the manufacturer’s protocol. RT-qPCR was performed using an ABI PRISM 7700 sequence detection system instrument and software (Applied Biosystems, Foster City, CA, USA). RT-qPCR assays of eight globin transcripts were performed using SYBR^®^ Select Master Mix (Applied Biosystems) with primers and PCR conditions described previously (Tangprasittipap et al., 2015). Absolute globin gene transcript copy numbers were calculated by comparison with standard curves generated from a plasmid DNA encoding each globin template. The transcript levels of other genes identified by microarray as differentially expressed between FL- and AB-derived erythroblasts were measured using the Assays-on-Demand Gene Expression Products (Applied Biosystems) according to the manufacturer’s instructions. The PCR primers are listed in Table S1. Expression data for each transcript were normalized to the reference transcript 18S ribosomal RNA. The relative fold changes were analyzed by the 2^−ΔΔCt^ method (Livak & Schmittgen, 2001).

### Microarray data analysis

Statistical analysis of microarray data was performed using R version 3.4.1 (R Core Team, 2017). The Affymetrix raw data files (.CEL files) were preprocessed using the robust multi-array average (RMA) method (Irizarry et al., 2003) implemented in the oligo package version 1.40.2 for background correction, log-transformation, and quantile normalization. Differential gene expression between FL- and AB-derived erythroblasts was determined using moderated t-statistics (Smyth, 2004) implemented in the Limma package version 3.32.10. The resulting *p*-values were further adjusted for multiple testing to minimize the number of false positives in the study using the Benjamini & Hochberg False Discovery Rate (FDR) method (Benjamini & Hochberg, 1995). Differentially expressed transcripts were defined as transcripts with an absolute fold change of at least 1.50 and the adjusted *p*-value less than 0.05. Hierarchical clustering (single linkage) of these transcripts was employed to group genes with similar expression patterns across samples using MeV version 4.9 (Saeed et al., 2003). The microarray data are available from the NCBI Gene Expression Omnibus database under accession number GSE109186. Microarray data of culture day 7 published previously (Xu et al., 2012) were downloaded from the GEO database and analyzed as described above. Meta-analysis of the two studies combined into one dataset was performed by the Rank Product method using the R package RankProd version 3.2.0 (Hong et al., 2006) based on default parameters. Only genes represented on both microarray platforms (Xu et al. study, Affymetrix human genome U133 Plus 2.0 array; our study, Affymetrix Human Gene 2.0 ST Array) were considered for meta-analysis. In each study, for multi-probe genes, the probe with the highest interquartile range (IQR) was selected to represent the gene to be used in meta-analysis as recommended in (Gentleman, 2005; Walsh et al., 2015).

### Functional and pathway analyses

The functions and the associated pathways of all differentially expressed genes were analyzed according to information from the Gene Ontology (GO) database (Ashburner et al., 2000; Gene Ontology Consortium, 2015) and the Kyoto Encyclopedia of Genes and Genomes (KEGG) pathway database (Kanehisa & Goto, 2000). Functional enrichment analysis was performed using the ToppFun feature in the ToppGene Suite (Chen et al., 2009) using the cutoff of *p*-value = 0.05 with FDR correction. Lists of genes with significantly higher expression in FL- or AB-derived erythroblasts were used as input for ToppFun to identify pathways and GO terms over-represented among genes in each list. The selected pathways were displayed using Pathview (Luo & Brouwer, 2013; Luo et al., 2017). Significant GO terms were summarised using the REVIGO tool (Supek et al., 2011) with default settings.

### Gene regulatory network construction

To describe the transcriptional regulation of genes co-upregulated either in FL or AB-derived erythroblasts, transcription factors (TFs) that target these genes were identified and the gene regulatory network displaying putative interactions among TFs and their direct target genes was built using iRegulon (Janky et al., 2014). Specifically, regulatory sequences in the vicinity of each gene in the set were analyzed to detect the enriched TF binding motifs or ChIP-seq peaks from databases. Then, these signals were associated with candidate transcription factors and their optimal target sets were determined. The regulatory search regions were set to be 20 kb flanking the transcription start site (from −10 kb to +10 kb) and the enrichment score threshold was 3.0. Other parameters were set as default. The resulting networks were displayed using cytoscape version 3.6 (Cline et al., 2007).

## Results

### *In vitro* culture of primary human fetal and adult erythroid progenitors

**Figure 1:**
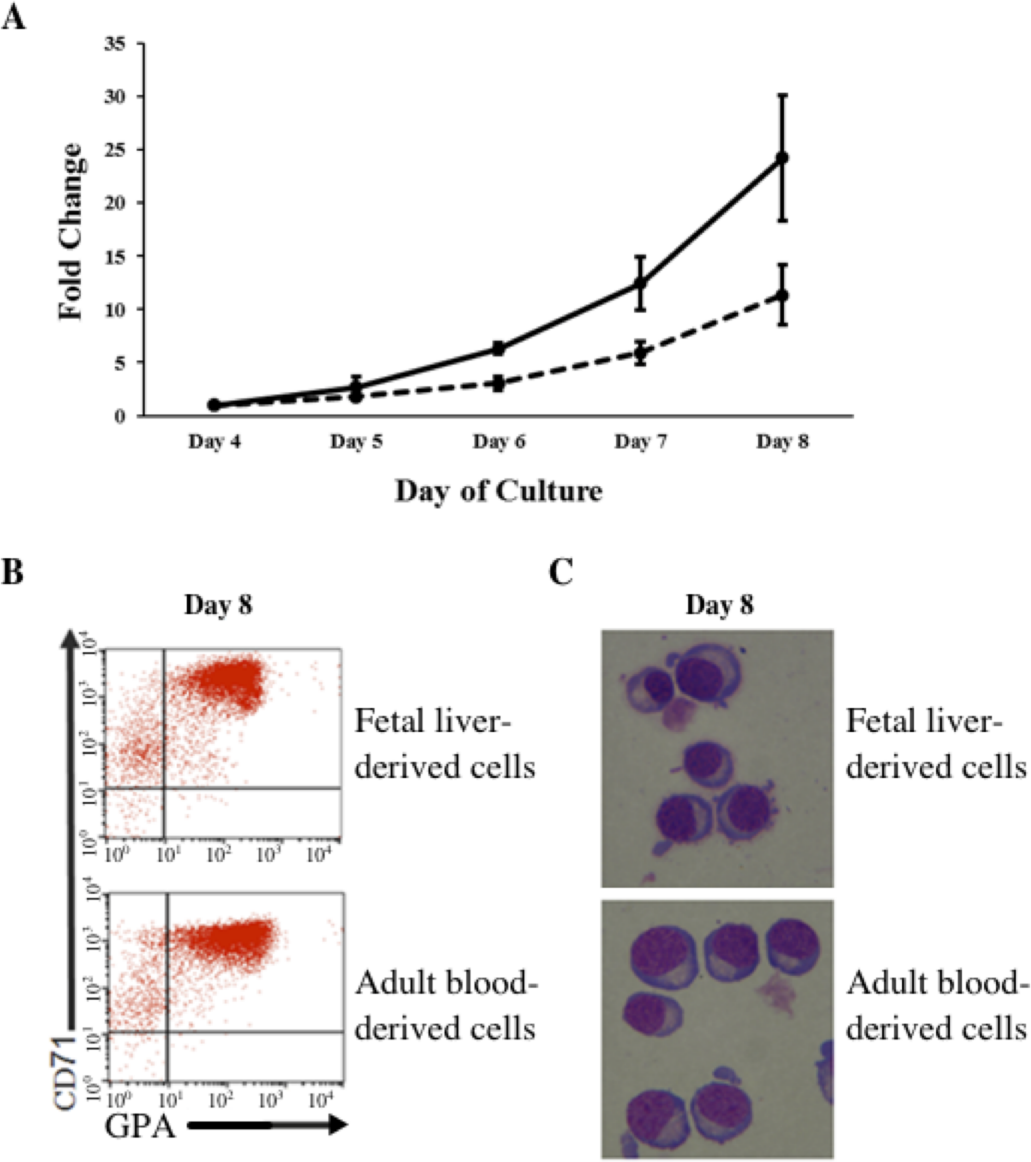
Amplification and maturation of CD34^+^ fetal liver (FL) and adult peripheral blood (AB) derived cells during *ex vivo* erythroid differentiation. (A) CD34^+^ immuno-enriched stem cells (FL, solid line; AB, dashed line) were grown in liquid culture according to the two-step protocol. Note that the purity of immuno-enriched cells was not assessed before expansion. Total cells were counted daily from day 4 to day 8 of culture. Mean values of fold-change relative to day 4 of culture with the standard deviation for five experiments are shown. (B) Flow cytometry analysis for transferrin receptor (CD71) and glycophorin A (GPA) surface expression on culture day 8. Flow cytometric gates are denoted as CD71^−^/GPA^−^ (lower left quadrant), CD71^+^/GPA^−^ (upper left quadrant), CD71^+^/GPA^+^ (upper right quadrant) and CD71^−^/GPA^+^ (lower right quadrant). (C) Morphology of cells harvested at culture day 8.

*In vitro* differentiated erythroid progenitors were grown from FL- and AB-derived CD34^+^ cells. A greater amplification of erythroid cells was observed from FL than AB cells in our culture system (Fig. 1A). After day 8 of culture, the majority of cells had differentiated into erythroid cells, as shown by the flow cytometric gating of CD71^+^/GPA^+^ cells (Fig. 1B) and morphology (Fig. 1C). The distinctive properties of FL- and AB-derived differentiated erythroblasts with respect to globin expression were assessed by quantification of absolute levels of globin transcripts on day 8 cultured cells (Table 1). The FL-derived erythroblasts demonstrated markedly elevated expression of the fetal *γ*-globin gene, together with elevated expression of embryonic globin genes (ε- and *ζ*-globin) compared with AB-derived erythoblasts. In contrast, the levels of adult *β*-globin and *δ*-globin transcripts were markedly higher in AB-derived erythroblasts. Transcript levels of the *α*-, µ-, and *θ*-globin genes were similar in both cell types. Hemoglobin analysis of day 14 cultured cells (Fig. S1) confirmed the predominant HbF in FL-derived erythroblasts, with a much lower HbF level in AB-derived erythroblasts (FL = 95.4 ± 1.5% vs. AB = 4.4 ± 0.2%). Neither HbZ nor HbE1 embryonic globins were detected by HPLC analysis. These data show that our *in vitro* culture system is a suitable model for comparing gene expression profiles during human erythroid development at fetal and adult stages.

**Table 1:** Absolute transcript levels of globin genes in the beta- and alpha-like globin clusters determined by reverse-transcription quantitative PCR in day 8 human fetal liver- and adult peripheral blood-derived cultured erythroblasts.

**Globin mRNA**	**Fetal liver**	**Adult blood**	*p*-value^b^
**Beta-like globin cluster (copies/ng total RNA)**^a^			
ε-globin	2.5 ± 1.2 ×10E3	7.8 ± 8.2 ×10E1	0.00922
*γ*-globin	1.2 ± 0.2 ×10E6	7.4 ± 1.3 ×10E4	0.00013
*δ*-globin	3.9 ± 0.9 ×10E1	1.1 ± 0.2 ×10E5	0.00031
*β*-globin	8.2 ± 3.7 ×10E4	1.7 ± 0.5 ×10E6	0.00135
**Alpha-like globin cluster (copies/ng total RNA)**^a^			
*ζ*-globin	4.5 ± 1.6 ×10E4	5.7 ± 5.3 ×10E1	0.00287
*μ*-globin	2.0 ± 0.5 ×10E4	2.2 ± 0.3 ×10E4	0.47716
*α*-globin	1.1 ± 0.1 ×10E6	9.7 ± 2.5 ×10E5	0.44619
*θ*-globin	4.1 ± 1.3 ×10E2	4.0 ± 1.1 ×10E2	0.88345

**Notes.**

a Average absolute copy numbers were estimated from standard curves generated from plasmid DNA; errors represent standard deviation from five experiments.

b The *p*-values represent the levels of statistical difference between transcript levels of globin genes between fetal liver- and adult peripheral blood-derived erythroblasts, which were calculated using Welch’s *t*-test.

### Comparative gene expression analysis of erythroblasts derived from fetal liver and adult blood

**Figure 2:**
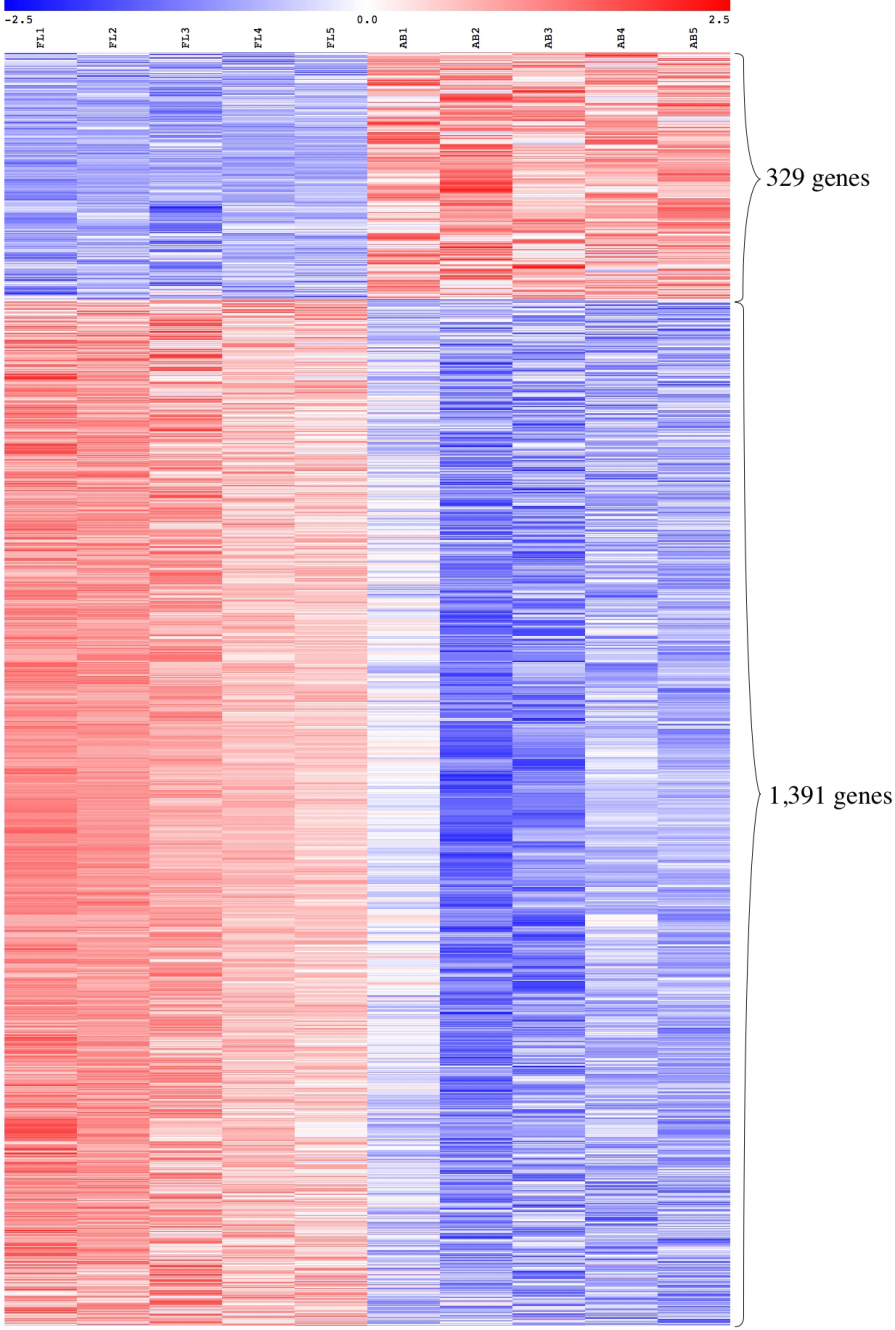
Expression profiles of differentially expressed transcripts in fetal liver (FL) compared with adult peripheral blood (AB) derived-erythroblasts. Heat maps show expression levels of differentially expressed transcripts among samples collected in this study. The gene expression level (row Z-score) is indicated by the red-blue color scale from high to low expression levels. Differentially expressed transcripts were determined with an absolute fold change ≥1.5 and the adjusted *p*-value < 0.05. FL1 to FL5, FL derived-erythroblast samples; AB1 to AB5, AB derived-erythroblast samples.

We performed global gene expression profiling to characterize stage-specific gene expression patterns during human erythroid development. Gene expression profiles of FL- and AB-derived erythroblasts were determined from five independent samples as biological replicates by oligonucleotide microarray experiments. 1391 genes (1,414 transcripts) expressed higher in FL compared with AB erythroblasts, whereas 329 genes (340 transcripts) showed the opposite pattern (Fig. 2, Table S2). With the exception of globin genes and genes with unknown function, the neuropilin (NRP) and tolloid (TLL)-like 2 gene (NETO2) is the most significant FL up-regulated gene (adjusted *p*-value = 1.1E−06, fold change = 9.53) while tenascin XB (TNXB) is the most significant AB up-regulated gene (adjusted *p*-value = 1E−05, fold change = 2.98). When non-globin genes with known function were ranked by fold change instead, secreted phosphoprotein 1 (SPP1) was the top FL up-regulated gene (adjusted *p*-value = 2.61E−03, fold change = 14.96) while carbonic anhydrase I (CA1) was the top AB up-regulated gene (adjusted *p*-value = 1.36E−04, fold change = 10) (Table S2).

The expression patterns of the globin gene family were examined as positive controls to validate the microarray platform for global comparison of FL- and AB-derived erythroblast transcriptomes. The *β*-globin (HBB) gene was identified as one of the top-ten genes with significantly higher expression in AB compared with FL erythroblasts (fold change = 34.33, adjusted *p*-value = 4.25E−05) (Table S2), in agreement with RT-qPCR data. In addition, the expression patterns of other globin genes are concordant with RT-qPCR data, including the *γ*-globin gene (HBG1) which showed a significantly higher expression level in the FL group (fold change = 2.89, adjusted *p*-value = 6.29E-03). The *ζ*-globin (HBZ) and ε-globin (HBE1) genes also had significantly higher expression in the FL group (Table S2).

### Functional analysis of differentially expressed genes

Toppfun functional enrichment analysis was performed on pathways and gene ontology (GO) terms represented among annotated functions of genes with significantly different expression between FL- and AB-derived erythroblasts in order to understand the biological significance of these genes (Table S3). More pathways were significantly associated with genes up-regulated in FL than AB (239 and seven pathway terms from various annotation data sources, respectively) (Table S3). In addition, more GO terms were revealed for FL than AB up-regulated genes, e.g., 457 and 28 enriched GO biological process terms, respectively. Overall, the enriched GO biological process terms of FL up-regulated genes summarized by REVIGO (Supek et al., 2011) indicate gene functions mainly associated with ncRNA metabolism, ribonucleoprotein complex biogenesis, cell cycle, and RNA localization, whereas the enriched terms among AB up-regulated genes indicate gene functions associated with regulation of immune response, transport, and purine deoxyribonucleoside metabolism. The top biological process GO terms were ribonucleoprotein complex biogenesis and one-carbon compound transport for FL and AB up-regulated genes, respectively. The top molecular function GO terms were RNA binding and IgE binding for FL and AB up-regulated genes, respectively. The top cellular component GO terms were ribonucleoprotein complex and side of membrane for FL and AB up-regulated genes, respectively.

### Transcriptional regulation networks of differentially expressed genes

**Figure 3:**
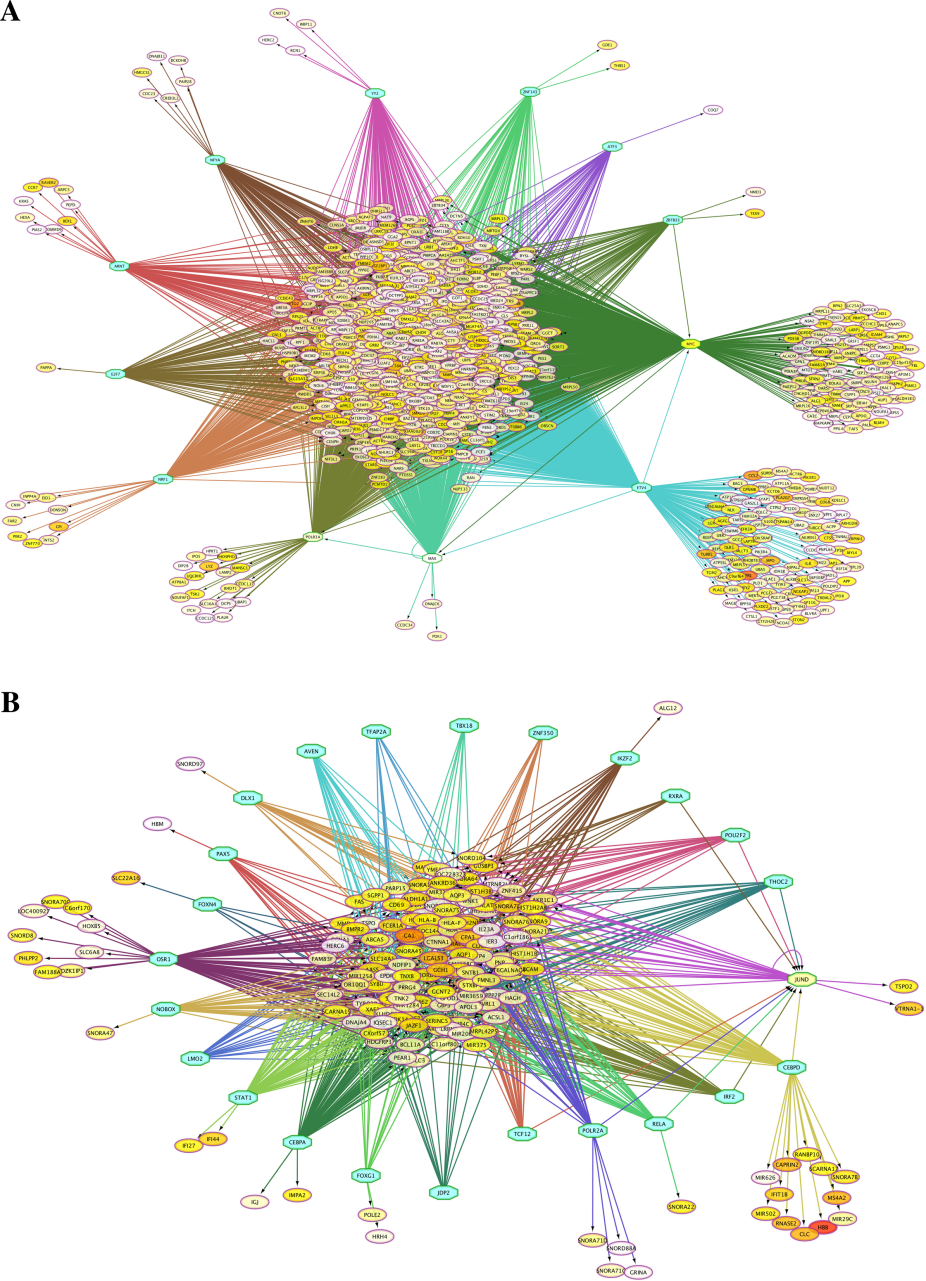
Transcription factor (TF)-gene regulatory network for genes differentially expressed between fetal liver (FL) and adult peripheral blood (AB) erythroblasts. Genes identified with significant differential expression between FL- and AB-derived cells from data generated in this study were used as input for constructing gene regulatory networks with iRegulon. (A) FL up-regulated target genes. (B) AB up-regulated target genes. The light blue octagons represent transcription factors and the ellipses represent target genes. Each ellipse is heat map color-coded by the degree of expression difference between FL and AB groups from 1.5 (white), 2 (yellow) to 6 (red) fold change. Interactions among TFs and target genes predicted by iRegulon are shown by connecting edges, and regulons for each TF are represented by different edge colors.

Because the switching of globin gene expression between fetal and adult stages is a transcriptionally controlled process, we identified putative transcription factor (TF) binding sites in the vicinity of genes identified by microarray analysis as differentially expressed between FL- and AB-derived erythroblasts (Table 2). Interactions among TFs and their target sites were revealed, in which TF-gene network connections showed largely common target genes among different TFs (Fig. 3). Some of these enriched TFs were also expressed differently between FL and AB, with expression profiles consistent with their target genes, i.e., TFs and their targets were up-regulated in the same cell type. FL up-regulated TFs included MYC, MAX, POLR3A and GABPA, whereas JUND was AB up-regulated. Among the FL up-regulated TFs, MYC was the top regulator with 793 potential targets among FL up-regulated genes.

**Table 2:** List of transcription factors (TF) predicted to target genes differentially expressed between fetal liver (FL) and adult peripheral blood (AB) erythroblasts from microarray data generated in this study.

TF	NES^a^	# targets (genes)	#motifs/tracks
FL up-regulated genes:			
ETV4	5.523	762	85/0
MYC	4.363	793	0/6
ATF3	4.189	46	2/0
ZBTB33	4.219	73	4/0
E2F7	4.100	116	8/0
ZNF143	3.961	78	2/0
NRF1	3.366	187	1/0
MAX	3.271	327	0/2
ARNT	3.299	88	5/0
YY2	3.501	112	2/0
POLR3A	3.034	322	1/0
NFYA	3.345	95	4/0
AB up-regulated genes:			
AVEN	4.307	22	4/0
POLR2A	4.293	30	0/4
THOC2	4.198	25	1/0
STAT1	4.087	58	5/0
JUND	4.026	24	0/2
POU2F2	4.019	23	0/2
NOBOX	3.888	25	0/2
TCF12	3.895	25	0/2
DLX1	3.721	19	0/1
RELA	3.677	35	3/0
CEBPA	3.640	60	10/0
LMO2	3.596	17	2/0
OSR1	3.518	74	5/0
RXRA	3.466	9	0/1
ZNF350	3.432	11	1/0
IKZF2	3.429	24	2/0
IRF2	3.161	42	5/0
PAX5	3.280	19	2/0
CEBPD	3.285	60	0/2
FOXG1	3.137	13	1/0
TBX18	3.082	8	1/0
JDP2	3.041	13	1/0
TFAP2A	3.027	9	1/0
FOXN4	3.017	6	1/0

**Notes.**

a NES (Normalized Enrichment Score): enrichment score of the motif or the maximal enrichment score for a given TF.

### Meta-analysis with previous microarray study

We reanalyzed the microarray data from Xu et al. (2012), in which a similar comparative study of FL- and AB-derived erythroblasts was performed. We did not include the data reported in Yang et al. (2013) for reanalysis or comparison, since only one sample of each cell type was studied on a different platform (RNA-seq). Moreover, studied cells in the Yang et al. were in a later stage of erythroblast maturation, which could bias the comparison of gene expression profiles. From the Xu et al. data, 420 genes (533 transcripts) were found to be FL up-regulated whereas 582 genes (790 transcripts) were AB up-regulated. This is fewer than reported in the original study (1,057 and 916 up-regulated genes in FL and AB cells, respectively; Table S1 of Xu et al.), suggesting that our analysis method is more conservative. A total of 18,965 genes were identified with features represented on both microarray platforms (Xu et al., Affymetrix human genome U133 Plus 2.0 array; our study, Affymetrix Human Gene 2.0 ST Array). A number of significant genes were identified only in each individual study (237 genes in ours and 50 genes in the Xu et al. study) owing to the presence of different features unique to each microarray platform (Table S2).

**Figure 4:**
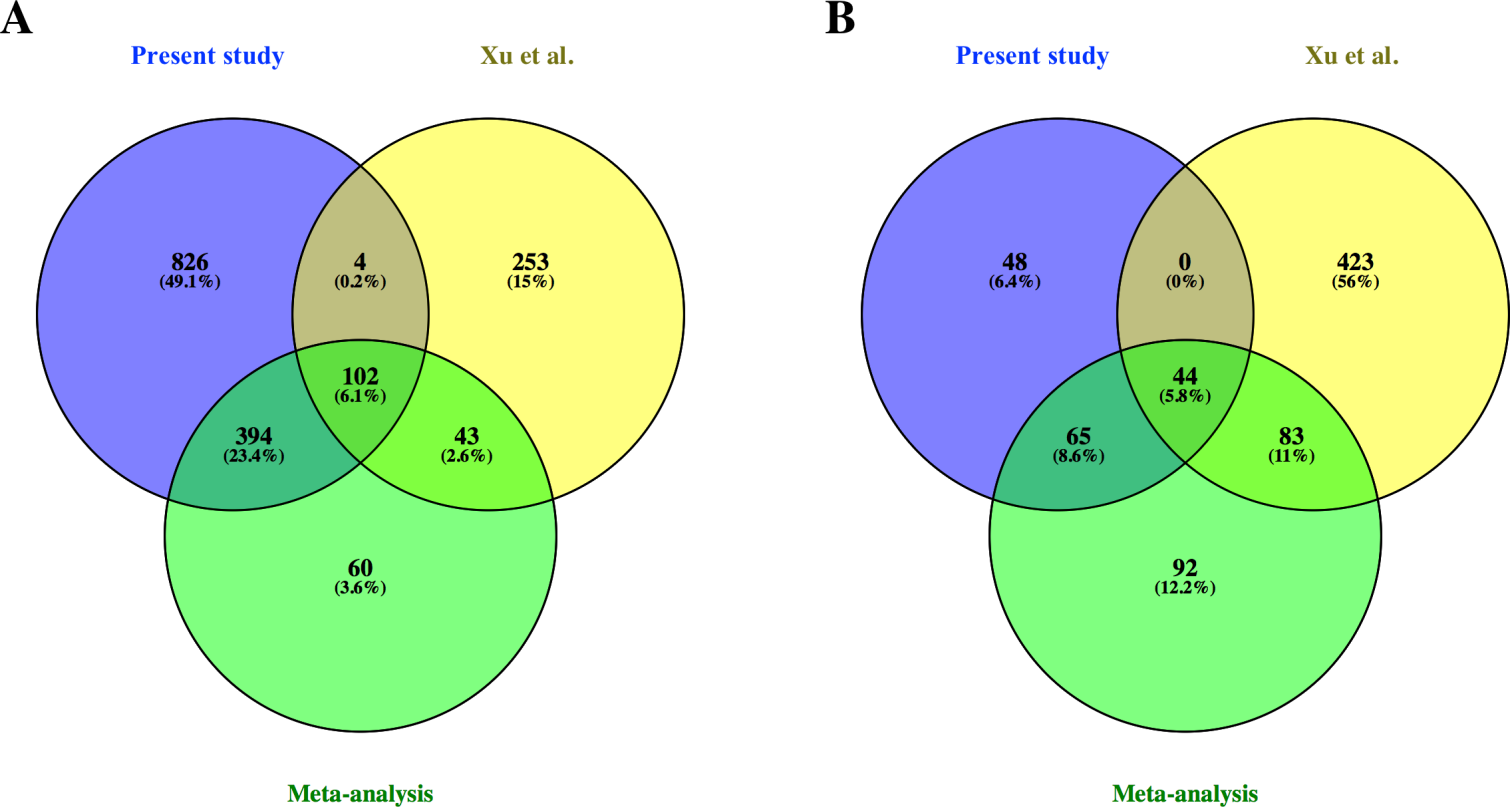
Comparison of differentially expressed genes identified in the present study, the Xu et al. (2012) study and meta-analysis of combined data. Venn diagrams show overlaps of genes detected as (A) fetal liver (FL) up-regulated and (B) adult peripheral blood (AB) up-regulated genes from the present study data analyzed separately, the Xu et al. (2012) study data analyzed separately, and meta-analysis of the combined dataset from both studies.

**Figure 5:**
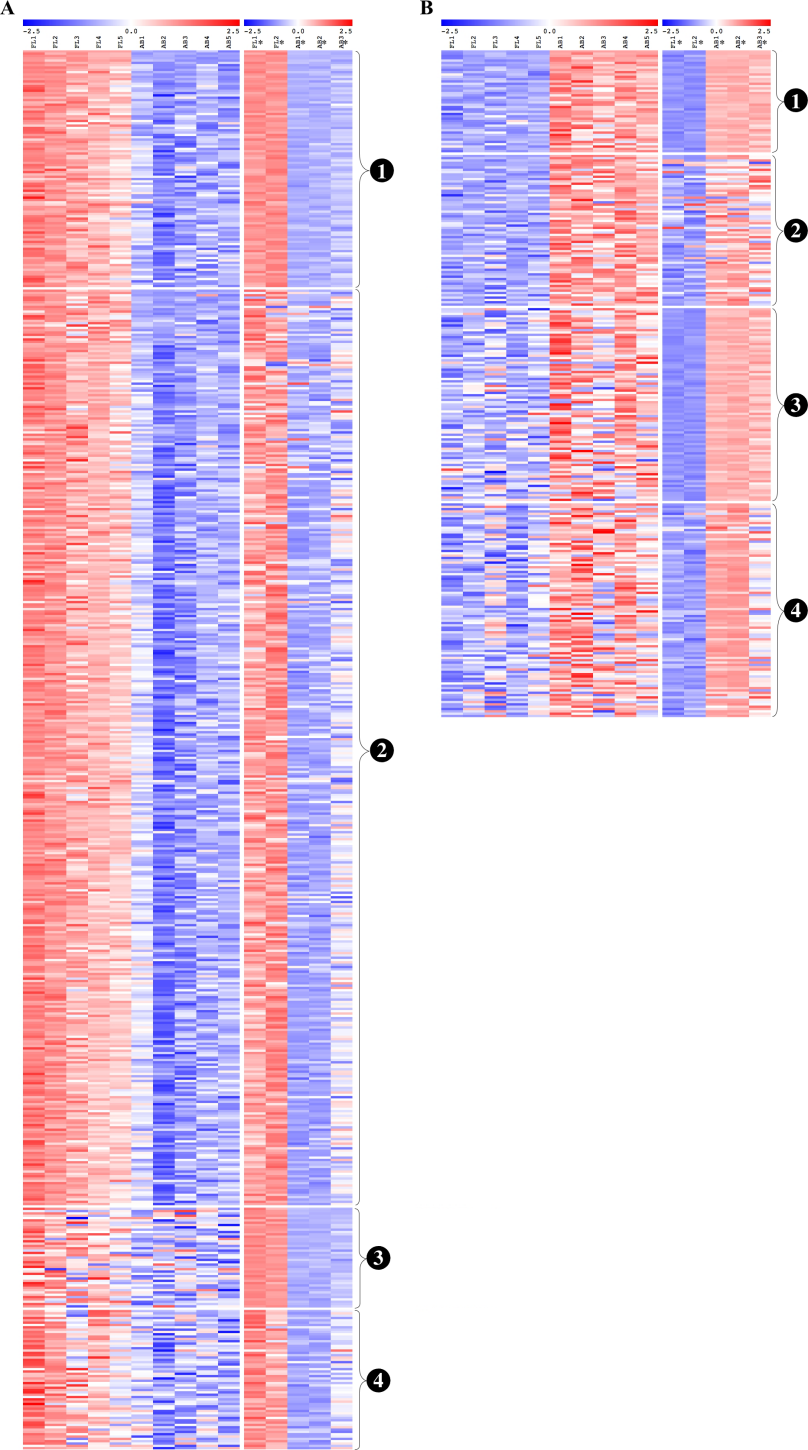
Reproducibility of differential gene expression patterns between the present study and the Xu et al. (2012) study. Heat maps show expression levels of significant differentially expressed genes from analysis of data collected for the present study and from the Xu et al. (2012) study. The expression level (row Z-score) is indicated by the red-blue color scale. For (A) fetal liver (FL) up-regulated and (B) adult peripheral blood (AB) up-regulated genes, genes are grouped as shown by the labels: (1) genes significant in both datasets analyzed separately and in meta-analysis of the combined dataset, (2) genes significant in the present study analyzed separately and in meta-analysis of the combined dataset, (3) genes significant in the Xu et al. (2012) dataset analyzed separately and in meta-analysis of the combined dataset, (4) genes not significant in either dataset when analyzed separately, but called significant by meta-analysis of the combined dataset. FL1 to FL5, FL-derived erythroblast samples in the present study; AB1 to AB5, AB-derived erythroblast samples in the present study; FL1* to FL2*, FL-derived erythroblast samples in the Xu et al. (2012) study; AB1* to AB3*, AB-derived erythroblast samples in the Xu et al. study.

From the common genes represented on both microarray platforms, a meta-analysis of the combined dataset from both studies was performed. A total of 599 FL up-regulated and 284 AB up-regulated genes, respectively, were identified by meta-analysis (Table S4). These genes were compared with significant genes from each study dataset analyzed separately. 102 and 44 genes were identified as FL up-regulated or AB up-regulated, respectively in both studies, irrespective of whether the datasets were analyzed separately or combined for meta-analysis (Fig. 4). The combined dataset showed 60 FL up-regulated and 92 AB up-regulated genes in meta-analysis, which were not significant for either dataset analyzed separately (Fig. 4). The finding of 152 genes with significant difference in expression only in meta-analysis indicates that combining datasets gives increased power to detect genes with reproducible changes in expression between the two studies. The heat map representations of meta-analysis significant genes further illustrate the increased power of meta-analysis to detect genes with reproducible patterns (Fig. 5). Despite the increased power to detect reproducible patterns of gene expression by meta-analysis, many genes showed significant changes in expression only in one microarray study dataset analyzed separately, and not the other or the combined dataset in meta-analysis (Fig. 4; Fig. S2).

Although reproducible patterns of gene expression were identified by meta-analysis, the discrepancies between differentially expressed genes identified from our data and those from the Xu et al. study data led us to perform functional enrichment analysis of the combined dataset to determine which pathways/biological processes are reproducible between studies (Table S5). More pathways were significantly associated with genes up-regulated in FL than AB (76 and 29 pathway terms, respectively), although more AB pathways were identified in the combined dataset than from our data analyzed separately (Table S5). Similarly, more GO terms were revealed for FL than AB up-regulated genes, e.g., 268 and 34 enriched GO biological process terms, respectively. Among FL up-regulated genes in the combined dataset, the summary of GO biological process terms was similar to the analysis results of our data (ncRNA metabolism and ribonucleoprotein complex biogenesis) with some additional terms (response to nitrogen compound and glucose catabolism). For AB up-regulated genes, the summarized GO biological process terms from the combined dataset were quite different from our dataset (regulation of cell differentiation, response to oxidative stress) although transport process was in common. The top enriched biological process and molecular function GO terms were the same as our data analyzed separately (ribonucleoprotein complex biogenesis and RNA binding respectively), whereas mitochondrion was the top cellular component GO term. Among AB up-regulated genes in the combined dataset, the top enriched GO terms for each category were different from our data analyzed separately (positive regulation of cell differentiation, ammonium transmembrane transporter activity and nucleosome).

Analysis of TFs targeting meta-analysis significant genes showed some common TFs with the ones identified in significant genes from our microarray data analyzed separately. The common TFs targeting FL up-regulated genes were MYC, MAX, ZBTB33, ZNF143, and POLR3A, whereas the common TFs for AB up-regulated genes were CEBPA, CEBPD, IKZF2, POU2F2, and RELA (Table 2, Table S6 , Fig. S3). Among the FL up-regulated TFs, MYC and its TF partner MAX (Grandori et al., 2000) were the top regulators with 361 and 370 potential target genes with FL up-regulated expression.

### Validation of meta-analysis differentially expressed genes by reverse-transcription qPCR

To validate the findings from microarray experiments, the same FL and AB RNA samples as used in microarray were recruited for RT-qPCR. RT-qPCR experiments were performed for thirteen genes with significant differential expression by microarray in our dataset analyzed separately and in meta-analysis of the combined dataset. Five FL up-regulated genes (IGF2BP3, BCAT1, CHD7, NETO2, MYC) and seven AB up-regulated genes (CA1, LGALS3, FAS, MAP3K5, TNXB, JUND, JAZF1) were confirmed (Table 3). Differential expression of the LIN28B gene could not be assessed by RT-qPCR since the expression in adult erythroblasts was lower than the limit of detection (Ct greater than 35).

**Table 3:** Reverse-transcription quantitative PCR (RT-qPCR) validation results of differentially expressed genes identified by meta-analysis of the combined microarray dataset.

Gene symbol	Description	Microarray fold change^a^ (*p*-value)	Meta-analysis^b^ fold change (*p*-value)	qPCR fold change
Up-regulated genes in fetus (FL):				
LIN28B	lin-28 homolog B	4.75 [6.96E−06]	11.97 [1.11E−13]	ND^c^
IGF2BP3	insulin like growth factor 2 mRNA binding protein 3	4.50 [3.98E−06]	13.97 [1.45E−13]	430 ± 350
BCAT1	branched chain amino acid transaminase 1	4.09 [1.00E−05]	2.72 [2.91E−08]	5.5 ± 2.8
CHD7	chromodomain helicase DNA binding protein 7	3.68 [1.14E−04]	2.61 [1.01E−07]	8.1 ± 4.3
NETO2	neuropilin and tolloid like 2	9.53 [1.10E−06]	3.36 [1.37E−10]	6.6 ± 2.6
MYC	MYC proto-oncogene, bHLH transcription factor	2.11 [6.28E−03]	1.81 [2.23E−03]	2.0 ± 0.6
Up-regulated genes in adult (AB):				
CA1	carbonic anhydrase 1	10.00 [1.36E−04]	9.88 [1.97E−16]	180 ± 110
LGALS3	galectin 3	5.66 [2.23E−04]	5.28 [7.97E−13]	16 ± 4.0
JAZF1	JAZF zinc finger 1	3.05 [7.31E−03]	3.72 [3.37E−09]	13.41 ± 1.19
MAP3K5	mitogen-activated protein kinase kinase kinase 5	2.74 [1.09E−03]	2.84 [1.78E−08]	13 ± 2.6
FAS	Fas cell surface death receptor	2.33 [5.82E−04]	3.00 [4.32E−08]	10 ± 3.0
TNXB	tenascin XB	2.98 [1.00E−05]	1.58 [4.96E−06]	75 ± 46
JUND	JunD proto-oncogene, AP-1 transcription factor subunit	1.67 [2.44E−02]	1.29 [4.46E−03]	1.4 ± 0.6

**Notes.**

a Microarray data from this study analyzed using the Limma package.

b Meta-analysis of the data from this study combined with data reported in Xu et al. (2012) analyzed using the RankProd package.

c ND, Not determined as below the limit of detection in AB cell samples (Ct greater than 35).

### Common pathways associated with differentially expressed genes

**Figure 6:**
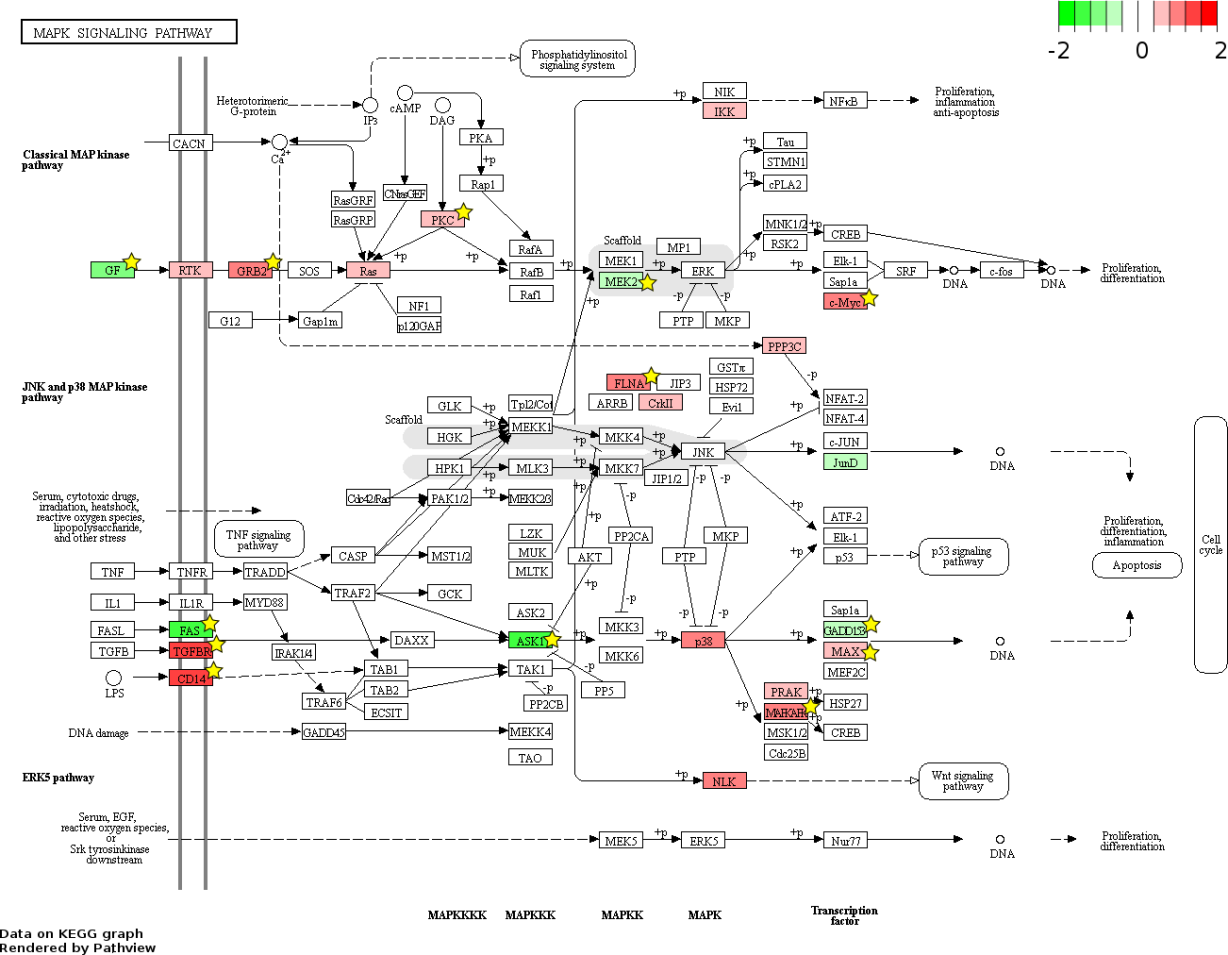
Fetal-adult differentially expressed genes mapped to the ‘MAPK signaling’ pathway. The KEGG pathway diagram (Kanehisa et al., 2012) shows the mapping of fetal liver (FL) up-regulated and adult peripheral blood (AB) up-regulated genes from data generated in this study to the ‘MAPK signaling’ pathway. The differentially expressed genes are heat map color-coded by the degree of expression difference from red (FL-up) to green (AB-up). Genes marked by yellow stars are also significant by meta-analysis of data generated in this study combined with that from the Xu et al. (2012) study.

**Figure 7:**
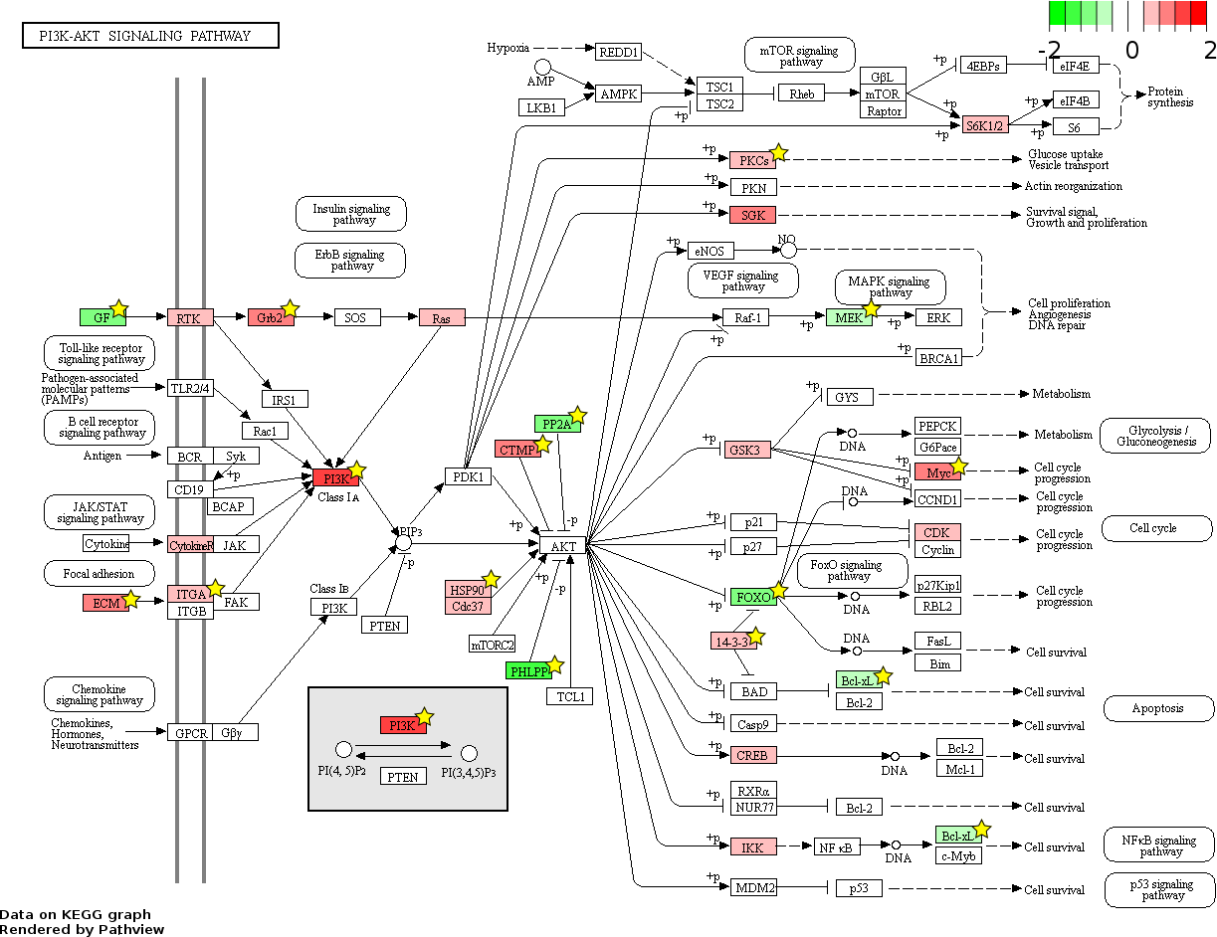
Fetal-adult differentially expressed genes mapped to the ‘PI3K-Akt signaling’ pathway. The KEGG pathway diagram (Kanehisa et al., 2012) shows the mapping of fetal liver (FL) up-regulated and adult peripheral blood (AB) up-regulated genes from data generated in this study to the ‘PI3K-Akt signaling’ pathway. The differentially expressed genes are heat map color-coded from red (FL-up) to green (AB-up). Genes marked by yellow stars are also significant by meta-analysis of data generated in this study combined with that from the Xu et al. (2012) study.

826 and 48 novel genes were identified only from data generated in this study to be FL up- and AB up-regulated, respectively (Fig. 4, Table S2). We explored the potential biological significance of these novel genes by inspection of KEGG pathways. We reasoned that novel genes with functions relevant to erythropoietic transition from the fetal to adult state would be connected in gene function pathways described previously in erythropoiesis. The mitogen-activated protein kinase (MAPK) and the phosphatidyl inositol 3 kinase (PI3K)-Akt pathways are activated in erythropoiesis (Hattangadi et al., 2011). Thirteen differentially expressed genes (CD14, GRB2, MYC, MAX, PRKCA, TGFBR1, MAPKAPK3, MAP3K5, MAP2K2, FAS, VEGFA, DDIT3, FLNA) were identified in the MAPK pathway from meta-analysis together with eleven novel genes from our data (CHUK, CRK, IGF1R, JUND, KRAS, NRAS, MAPK14, NLK, PPP3CA, MAPKAPK5, FGF5) (Fig. 6). Twenty differentially expressed genes (TNXB, COL4A5, SPP1, THBS1, VEGFA, GRB2, HSP90AB1, HSP90B1, ITGA5, ITGAV, MYC, PHLPP2, PIK3R1, PRKCA, PPP2R5B, THEM4, YWHAB, BCL2L1, FOXO3, MAP2K2) were identified in the PI3K-Akt pathway from meta-analysis together with twelve novel genes from our data (CDC37, CDK4, CHUK, CREB3L2, GSK3B, IFNAR1, IGF1R, KRAS, NRAS, RPS6KB1, SGK1, FGF5) (Fig. 7). The MAPK and PI3-Akt pathways overlap with other pathways, including the cell-cycle and cancer. Other differentially expressed genes from meta-analysis together with novel genes identified only from our data mapped to these pathways (Figs. S4 and S5).

## Discussion

The fundamental differences between fetal and adult erythropoiesis, including the hemoglobin switch are still rather poorly understood, as only two transcriptomic studies of erythroblasts derived from primary fetal liver have been conducted previously (Xu et al., 2012; Yang et al., 2013). The data obtained in this study provide further insight into the differences in gene expression between fetal and adult erythroblasts. We established new erythroblast cell cultures derived from FL and AB HSCs. The expansion and differentiation of erythroblasts in our culture system recapitulates the changes observed during *in vivo* human development, as shown by the markedly different patterns of embryonic, fetal and adult globins in the FL- compared with AB-derived erythroblasts. Although the *in vitro* cultured cells are suitable for modelling aspects of erythropoiesis *in vivo*, the observed differences between FL- and AB-derived erythroblasts could be confounded by heterogeneity of CD34^+^ HSCs subpopulations in the primary tissue samples. Until methods are available for isolation of pure HSCs and their long-term expansion *in vitro*, this caveat applies to all studies of cultured erythroblasts.

In addition to the globin genes, other genes with reproducible patterns of differential expression include known regulators of the hemoglobin transcriptional switch. The BCL11A gene is overexpressed in adult cells and acts as the major repressor of fetal *γ*-globin genes (Sankaran et al., 2008). BCL11A is significantly upregulated in AB-derived erythroblasts in both our and the Xu et al. datasets analyzed separately (Table S2), or combined in meta-analysis (Table S4). JAZF1, or TAK1-interacting protein 27 (TIP27) is another important regulator of hemoglobin switching found to be significantly upregulated in AB-derived erythroblasts in our dataset analyzed separately (Table S2), or combined in meta-analysis (Table S4). JAZF1 interacts with nuclear receptor TAK1 (TR4) (Nakajima et al., 2004), and TR4 is part of the DRED (direct repeat erythroid-definitive, TR2/TR4) complex. DRED is important for repression of *γ*-globin genes in adult cells (Cui et al., 2011; Tanabe et al., 2002; Tanabe et al., 2007).

Other genes implicated in hemoglobin switching show reproducible up-regulation in FL cells, including insulin-like growth factor 2 mRNA-binding protein 1 and 3 (IGF2BP1 and IGF2BP3) and LIN28B (Table 3; Table S4). Over-expression of these genes decreases *β*-globin expression in adult erythroblasts (De Vasconcellos et al., 2017). LIN28B over-expression in cultured adult erythroblasts reduces the expression of *let-7*, which in turn leads to increased HbF expression (Helsmoortel et al., 2016; Lee et al., 2013). The expression of genes marking the transition of fetal-to-adult erythropoiesis including CA1, GCNT2, and BCL11A are negatively regulated by LIN28B (Lee et al., 2013). This is consistent with the observation that these three genes are up-regulated in AB cells (Table S2, Table 3, Table S4). Some of the significant differentially expressed genes identified only in our data, such as CHD4, have functions in hemoglobin switching. CHD4 (Mi2*β*), a critical component of NuRD chromatin modifying complexes, was reported to mediate silencing of the fetal *γ*-globin gene in adult erythroid cells (Amaya et al., 2013).

As expected, the expression of globin genes and their regulators were shown to differ between FL- and AB-derived erythroid cells. The differences in expression of other genes not known to be directly related to globin gene expression could provide insights into the main physiological differences between FL- and AB-derived erythroblasts. Functional enrichment analysis of FL-upregulated genes in both our data and the combined dataset revealed ribonucleoprotein complex biogenesis as the top biological process term. The cytoplasmic and mitochondrial ribosomes constitute the major ribonucleoprotein complexes for protein synthesis. Proliferation of cells imposes a high demand for protein synthesis, requiring expression of genes for ribosomal proteins and non-coding RNAs. The *POLR3A* gene was identified as FL-upregulated and as a TF with many potential targets among FL-upregulated genes. *POLR3A* encodes the largest subunit of RNA Polymerase III (Pol III). Pol III transcribes small non-coding RNAs crucial for the translation machinery, such as 5S ribosomal RNA and all transfer RNAs. The regulation of growth and cell cycle control are intimately connected to Pol III activity (Dumay-Odelot et al., 2010) suggesting that FL-derived erythroid cells are engaged in a gene expression program geared more towards proliferation driven by a higher Pol III activity compared with AB-derived erythroid cells. The higher proliferation rate of FL-derived erythroblasts observed in culture (Fig. 1A) is consistent with this inference. However, confounding heterogeneity of HSCs (see above) cannot be excluded as a reason for the observed differences in proliferation rates. Notwithstanding this caveat, FL HSCs thus retain their intrinsic property of enhanced proliferation relative to AB HSCs in the *in vitro* culturing system, and differences in microenvironments between fetal and adult erythropoiesis *in situ* are not needed to explain the differences in cell behavior.

Further insights into the main physiological differences between FL- and AB-derived erythroblasts can be obtained from the transcriptional regulation networks of differentially expressed genes. The TF in the regulatory network of FL up-regulated genes with the most targets is MYC (Table 2), and the expression of this TF is also FL up-regulated (Table 3). The relevance of other TFs identified in the transcriptional networks of differentially expressed genes is more difficult to assess, as except for JUND, the genes coding for these TFs are not differentially expressed (Tables S2 and S4). MYC heterodimerises with MAX to form a transcriptional activator. The cellular expression of MYC generally correlates with the proliferation rate in different cell types, in which MYC/MAX transcriptionally activates genes in a number of signaling pathways (Grandori et al., 2000). The expression of MYC is tightly controlled during adult erythropoiesis, since misregulation of MYC expression leads to lymphoma and leukemia (Delgado & Leon, 2010). The higher expression of MYC and MAX in FL compared with AB erythroid cells (Table S4) is consistent with the high SCF hormone level in fetal liver tissues, in which SCF activates the PI3K-Akt pathway and induces expression of MYC (Hattangadi et al., 2011). MYC may thus be a central nexus in driving the proliferation-dominant program in fetal erythroid cells, and its expression must be downregulated for transformation to the adult erythropoietic program. The precise dosage of MYC is critical in determining the differentiation, proliferation, and survival of erythroid cells, as shown by ectopic expression studies in mouse fetal liver erythroid progenitors (Jayapal et al., 2010). We propose that once the level of MYC is below a certain threshold, global epigenetic restructuring of the genome takes place, allowing key molecular events to occur in the adult erythropoiesis program such as hemoglobin switching.

Among the genes identified as most significantly up-regulated in FL-derived erythroblasts, IGF2BP3 has recently been described as an ontogenic master switch that regulates megakaryocyte morphogenesis. Following knockdown of IGF2BP3 expression, fetal megakaryocytes are transformed to a growth-arrested, adult-like state (Elagib et al., 2017). No direct role in erythropoiesis has been attributed to the NETO2 and BCAT1 genes also up-regulated in FL-derived erythroblast cells. However, these genes are over-expressed in proliferating cancer cells (Oparina et al., 2012; Tonjes et al., 2013; Zhou et al., 2013), suggesting that they may also be important for maintaining the proliferation-dominant program of fetal erythroid cells. The TNXB gene identified as significantly up-regulated in AB-derived erythroblasts (Table S2; Table 3) is a member of the tenascin family of extracellular matrix glycoproteins. To our knowledge, no direct role in erythropoiesis or hemoglobin switching has been attributed to TNXB or JUND. However, TNXB modulates proliferation signalling pathways in different cell types (Valcourt et al., 2015) and JUND acts as a negative regulator of the cell cycle in different cell types (Mechta-Grigoriou, Gerald & Yaniv, 2001). TXNB and JUND expression may therefore be required for the transformation of fetal erythroid progenitors to the weaker proliferating adult state.

According to the expression of erythroid surface markers and cell morphology, we reasoned that the expression profiles of culture day 7 cells reported in Xu et al. (2012) would represent a similar stage of erythroblast maturity to the samples obtained in this study, such that reproducible patterns of expression could be found between the two studies. In addition to gene expression profiling, the Xu et al. study also explored the chromatin landscape of FL and AB erythroblasts, interrogating histone marks and transcription factor occupancies. The main conclusion from these data was that FL- and AB-specific enhancers fine-tune the expression of genes, explaining the modest fold-changes observed in differentially expressed genes between the two cell types (Xu et al., 2012). The meta-analysis of the combined microarray dataset revealed differentially expressed genes not found in either dataset analyzed separately, indicating that combining data from independent studies increased power to detect genes with modest fold-changes. Fewer than 100 genes showed more than three-fold average change in either direction in the combined dataset (Table S4). The greater power to detect genes with reproducible, differential expression should provide more insight into the fundamental biological differences between FL and AB erythroblasts.

Although meta-analysis identified more genes with reproducible patterns of expression than the simple intersection of signficant genes from separate analysis of individual microarray datasets, many genes found significant in one study were not reproduced in another (Fig. 4). The non-reproducible significant genes unique to each study can result from small sample sizes in both studies, i.e., statistical anomaly. Moreover, expression patterns of some genes may not be reproducible between studies owing to differences in experimental design, including microarray platform and *in vitro* cell culture conditions. The majority of genes found to be differentially expressed only from our data were FL up-regulated, whereas the majority genes differentially expressed only from the Xu et al. dataset were AB up-regulated. The pathway analysis showing representation of novel FL up-regulated genes in the data from this study (Figs. 6 and 7) suggests that the culture system we employed is better suited for FL-derived erythroid cells, although more study is needed to confirm this.

## Conclusions

We performed microarray gene expression profiling experiments of human FL- and AB-derived erythroblasts. Our data provide new insights into the differences in gene regulation between fetal and adult erythropoiesis. Analyses of gene function and transcriptional regulation networks of differentially expressed genes showed that proliferation is dominant during the fetal stage, which is driven by the greater expression of the transcriptional activator MYC. Among the differentially expressed genes, the IGF2BP3, NETO2, BCAT1, TXNB and JUND were identified as putative novel regulators of the transition from the fetal to the adult erythroid cell state.

##  Supplemental Information

10.7717/peerj.5527/supp-1 Figure S1 Hemoglobin content analysis of fetal liver (FL) and adult peripheral blood (AB) differentiated cells from culture day 14 (A) Flow cytometry analysis for transferrin receptor (CD71) and glycophorin A (GPA) surface expression. Flow cytometric gates are denoted as CD71^−^/GPA^−^ (lower left quadrant), CD71^+^/GPA^−^ (upper left quadrant), CD71^+^/GPA^+^ (upper right quadrant) and CD71^−^/GPA^+^ (lower right quadrant). (B) High-performance liquid chromatography (HPLC) analysis of CD71^+^/GPA^+^ sorted cells. The major hemoglobin peaks are labeled on each graph.

10.7717/peerj.5527/supp-2 Figure S2 Expression profiles of genes with non-reproducible patterns of differential expression between this study and the Xu *et al.* study Heat maps show expression levels of genes with significant differential expression genes only from the present study or the Xu *et al.* study data analyzed separately, but not significant in meta-analysis of the combined dataset. Gene expression level (row Z-score) is indicated by the red-blue color scale from high to low expression levels. For (A) fetal liver (FL) up-regulated and (B) adult peripheral blood (AB) up-regulated genes, genes are grouped as shown by the labels: (1) genes found significant only in the present study data, (2) genes found significant only in the Xu *et al.* data. FL1 to FL5, FL-derived erythroblast samples in the present study; AB1 to AB5, AB-derived erythroblast samples in the present study; FL1* to FL2*, FL-derived erythroblast samples in Xu *et al.* study; AB1* to AB3*, AB-derived erythroblast samples in the Xu *et al.* study.

10.7717/peerj.5527/supp-3 Figure S3 Transcription factor (TF)-gene regulatory network for meta-analysis significant genes Genes identified with significant differential expression between fetal liver (FL) and adult peripheral blood (AB)-derived cells from meta-analysis of the combined dataset from this study and the Xu *et al.* study were used as input for constructing gene regulatory networks with iRegulon. (A) FL up-regulated target genes. (B) AB up-regulated target genes. The light blue octagons represent TFs and the ellipses represent target genes. Each ellipse is heat map color-coded by the degree of expression difference between FL and AB groups from 1.5 (white), 2 (yellow) to 6 (red) fold change. Interactions among TFs and target genes predicted by iRegulon are shown by connecting edges, and regulons for each TF are represented by different edge colors.

10.7717/peerj.5527/supp-4 Figure S4 Fetal liver (FL) and adult peripheral blood (AB) differentially expressed genes mapped to the ‘Cell cycle’ pathway The KEGG pathway diagram (Kanehisa et al., 2012) shows the mapping of FL up-regulated and AB up-regulated genes from data generated in this study to the ‘Cell cycle’ pathway. The differentially expressed genes are heat map color-coded from red (FL-up) to green (AB-up). Genes marked by yellow stars are also significant by meta-analysis of data generated in this study combined with that from the Xu *et al.* study.

10.7717/peerj.5527/supp-5 Figure S5 Fetal (FL)-adult (AB) differentially expressed genes mapped to the ‘Pathways in cancer’ pathway The KEGG pathway diagram (Kanehisa et al., 2012) shows the mapping of FL and AB up-regulated genes from data generated in this study to the ‘Pathways in cancer’ pathway. The differentially expressed genes are heat map color-coded from red (FL-up) to green (AB-up). Genes marked by yellow stars are also significant by meta-analysis of data generated in this study combined with that from the Xu *et al.* study.

10.7717/peerj.5527/supp-6 Table S1 Information on PCR primers and probes used for quantitative reverse-transcription PCR

10.7717/peerj.5527/supp-7 Table S2 Genes with significant differential expression between fetal liver (FL) and adult peripheral blood (AB)-derived erythroblasts Gene lists are shown in separate tabs showing FL up-regulated and AB up-regulated genes from separate analysis of microarray datasets of the present study and Xu *et al.* study (Xu et al., 2012). For each gene, the fold-change and adjusted p-value from Limma analysis is shown together with information on whether it is found significant in the dataset of another study or in meta-analysis, and whether the gene is represented by feature(s) present only in the microarray platform used for that study.

10.7717/peerj.5527/supp-8 Table S3 Functional enrichment analysis of differentially expressed genes The list of enriched biological pathways and GO terms obtained from the ToppFun program using genes differentially expressed between fetal liver (FL) and adult peripheral blood (AB) erythroblasts is displayed separately for FL up-regulated and AB up-regulated gene groups. GO terms are separated into biological process (BP), molecular function (MF), cellular component (CC). The pathway/GO p-value and q-value are reported along with the list of genes associated with each pathway/GO term.

10.7717/peerj.5527/supp-9 Table S4 The list of meta-analysis significant genes The fold change values and p-values are shown for significant genes obtained from RankProd meta-analysis of the combined microarray dataset of this study and the Xu *et al.* study (Xu et al., 2012).

10.7717/peerj.5527/supp-10 Table S5 Functional enrichment analysis of meta-analysis significant genes The list of enriched biological pathways and GO terms obtained from the ToppFun program using the training sets of meta-analysis significant genes are shown for fetal liver (FL) up-regulated and adult peripheral blood (AB) up-regulated gene groups. GO terms are separated into biological process (BP), molecular function (MF), cellular component (CC). The pathway/GO p-value and q-value are reported along with the list of genes associated with each pathway/GO term.

10.7717/peerj.5527/supp-11 Table S6 List of transcription factors (TF) predicted to target fetal liver (FL) up-regulated and adult peripheral blood (AB) up-regulated genes called significant by meta-analysis

## Additional Information and Declarations

### Data Availability

The following information was supplied regarding data availability: The microarray data are available from the NCBI Gene Expression Omnibus database under accession number GSE109186.
