# Supplementary figures and images for: Comparison of gene expression profiles between human erythroid cells derived from fetal liver and adult peripheral blood
Source: PeerJ. 2018 Aug 31;6:e5527. doi: 10.7717/peerj.5527 (PMC6120446; doi:10.7717/peerj.5527)

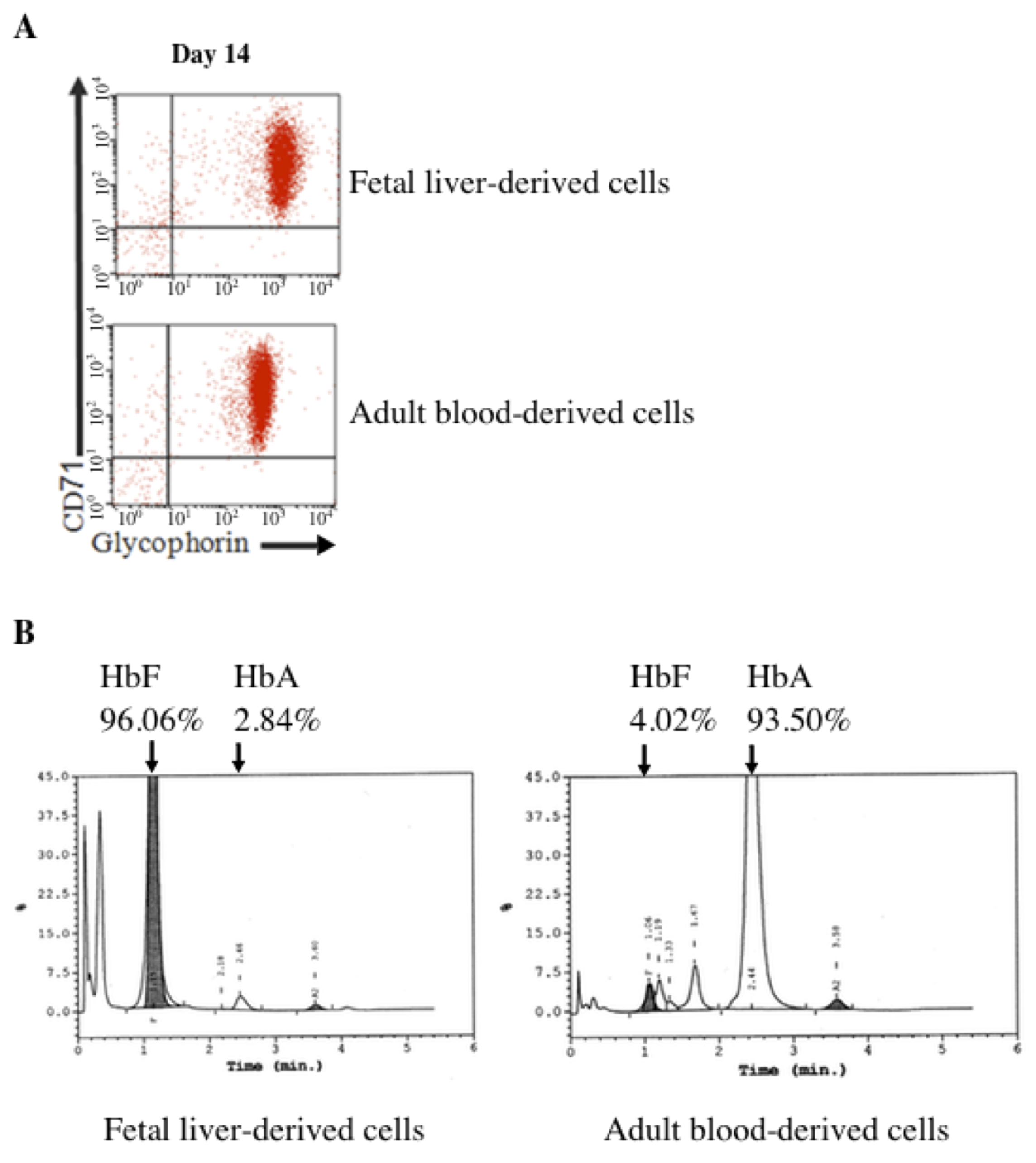

Supplement: Figure S1 — (A) Flow cytometry analysis for transferrin receptor (CD71) and glycophorin A (GPA) surface expression. Flow cytometric gates are denoted as CD71−/GPA− (lower left quadrant), CD71+/GPA− (upper left quadrant), CD71+/GPA+ (upper right quadrant) and CD71−/GPA+ (lower right quadrant). (B) High-performance liquid chromatography (HPLC) analysis of CD71+/GPA+ sorted cells. The major hemoglobin peaks are labeled on each graph. [file peerj-06-5527-s001.png]

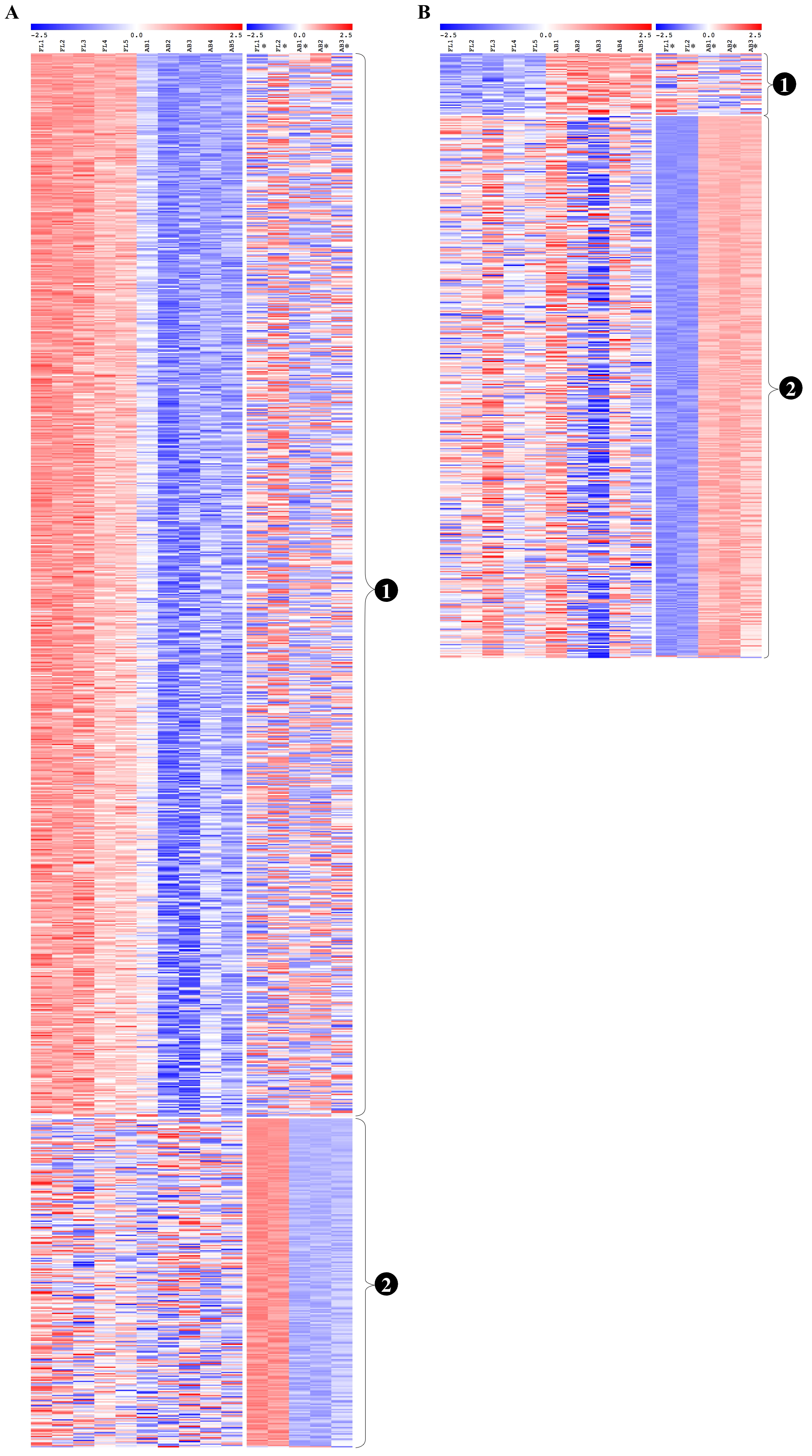

Supplement: Figure S2 — Heat maps show expression levels of genes with significant differential expression genes only from the present study or the Xu et al. study data analyzed separately, but not significant in meta-analysis of the combined dataset. Gene expression level (row Z-score) is indicated by the red-blue color scale from high to low expression levels. For (A) fetal liver (FL) up-regulated and (B) adult peripheral blood (AB) up-regulated genes, genes are grouped as shown by the labels: (1) genes found significant only in the present study data, (2) genes found significant only in the Xu et al. data. FL1 to FL5, FL-derived erythroblast samples in the present study; AB1 to AB5, AB-derived erythroblast samples in the present study; FL1* to FL2*, FL-derived erythroblast samples in Xu et al. study; AB1* to AB3*, AB-derived erythroblast samples in the Xu et al. study. [file peerj-06-5527-s002.png]

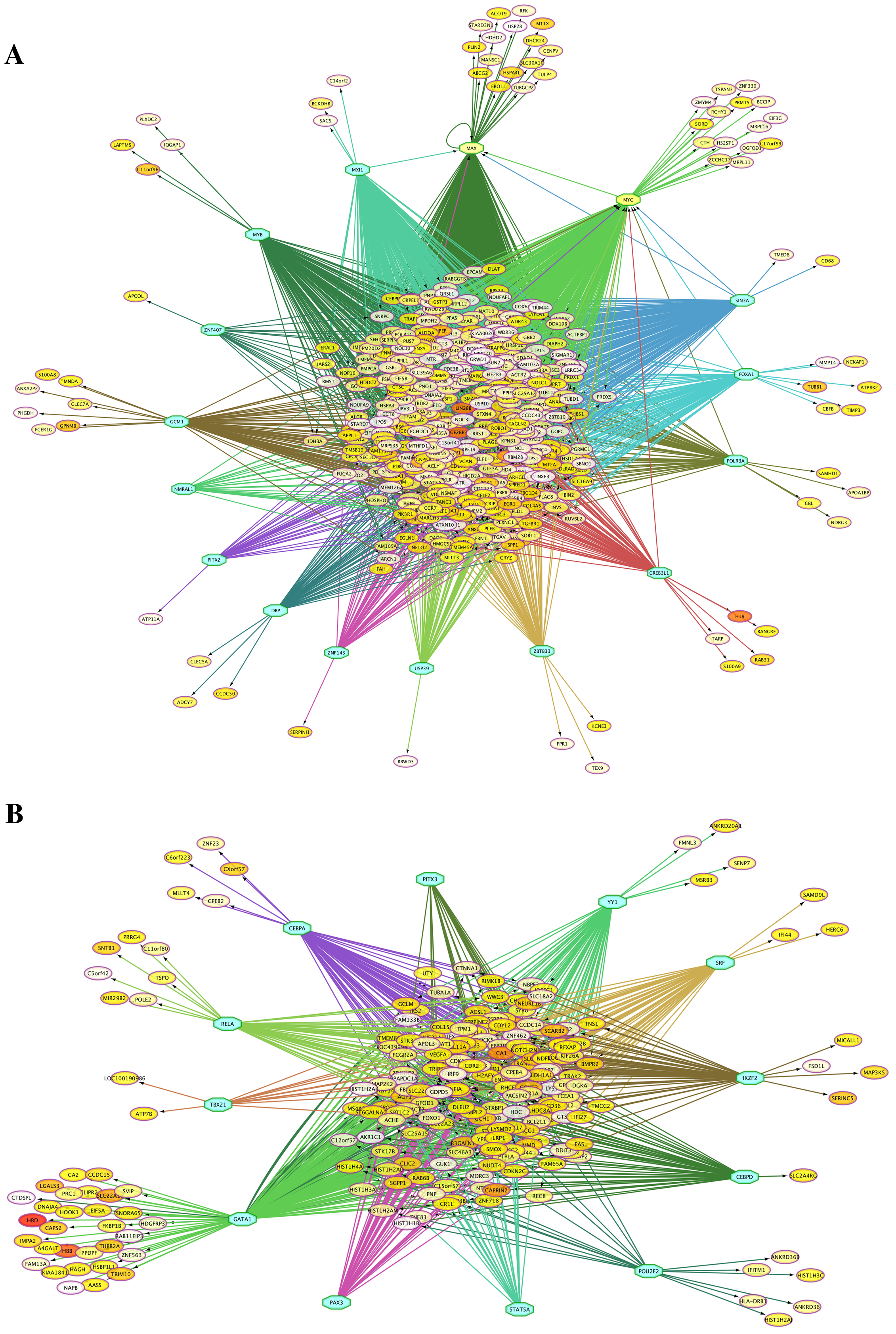

Supplement: Figure S3 — Genes identified with significant differential expression between fetal liver (FL) and adult peripheral blood (AB)-derived cells from meta-analysis of the combined dataset from this study and the Xu et al. study were used as input for constructing gene regulatory networks with iRegulon. (A) FL up-regulated target genes. (B) AB up-regulated target genes. The light blue octagons represent TFs and the ellipses represent target genes. Each ellipse is heat map color-coded by the degree of expression difference between FL and AB groups from 1.5 (white), 2 (yellow) to 6 (red) fold change. Interactions among TFs and target genes predicted by iRegulon are shown by connecting edges, and regulons for each TF are represented by different edge colors. [file peerj-06-5527-s003.png]

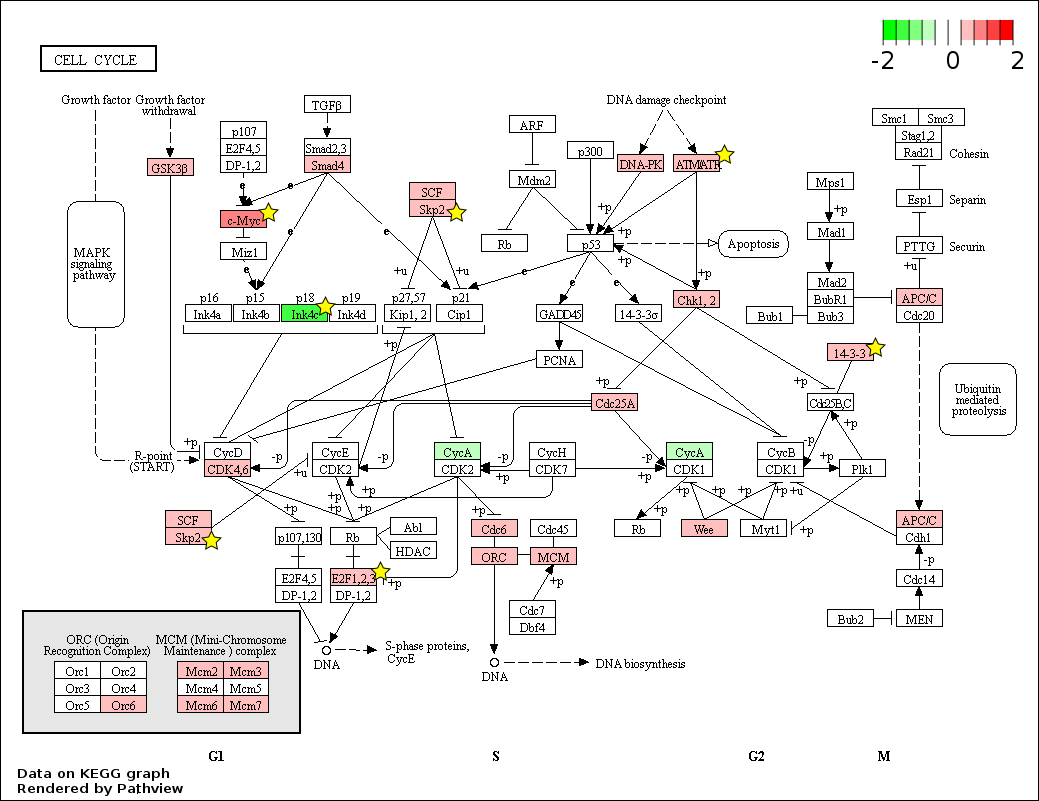

Supplement: Figure S4 — The KEGG pathway diagram (Kanehisa et al., 2012) shows the mapping of FL up-regulated and AB up-regulated genes from data generated in this study to the ‘Cell cycle’ pathway. The differentially expressed genes are heat map color-coded from red (FL-up) to green (AB-up). Genes marked by yellow stars are also significant by meta-analysis of data generated in this study combined with that from the Xu et al. study. [file peerj-06-5527-s004.png]

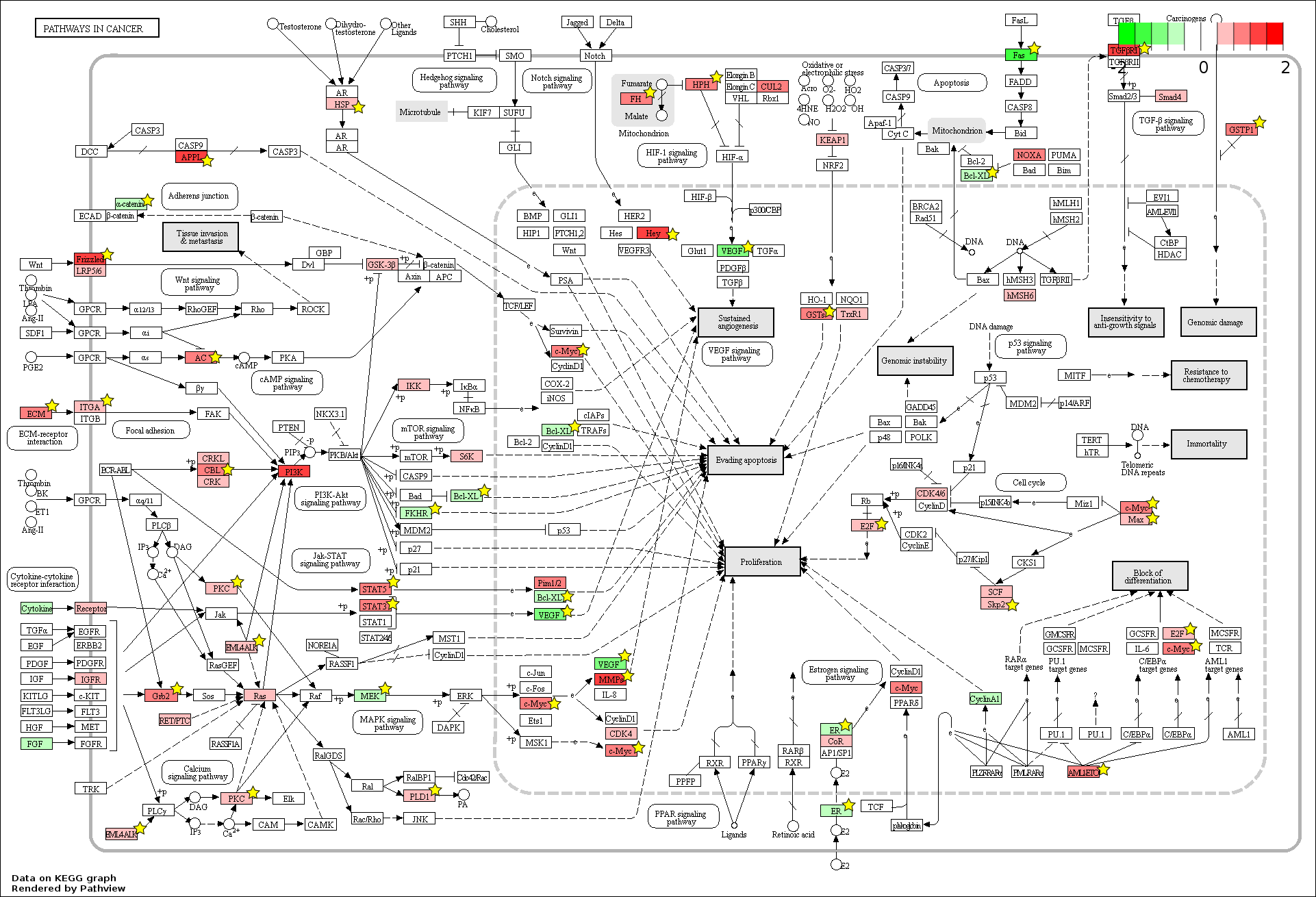

Supplement: Figure S5 — The KEGG pathway diagram (Kanehisa et al., 2012) shows the mapping of FL and AB up-regulated genes from data generated in this study to the ‘Pathways in cancer’ pathway. The differentially expressed genes are heat map color-coded from red (FL-up) to green (AB-up). Genes marked by yellow stars are also significant by meta-analysis of data generated in this study combined with that from the Xu et al. study. [file peerj-06-5527-s005.png]
