# Supplementary material for: Comparison of gene expression profiles between human erythroid cells derived from fetal liver and adult peripheral blood
Source: PeerJ. 2018 Aug 31;6:e5527. doi: 10.7717/peerj.5527 (PMC6120446; doi:10.7717/peerj.5527)
Supplement: Table S1 [file peerj-06-5527-s006.docx]

| Assays | Amplicons | Sequence (5’ to 3’) or TaqMan gene expression | Modification |
| --- | --- | --- | --- |
| Gene expression | CA1 | [Hs00266139_m1](https://www.thermofisher.com/taqman-gene-expression/product/Hs00266139_m1?CID=&ICID=&subtype=) | 5’Fam3’NFQ-MGB |
|  | LGALS3 | [Hs00173587_m1](https://www.thermofisher.com/taqman-gene-expression/product/Hs00173587_m1?CID=&ICID=&subtype=) | 5’Fam3’NFQ-MGB |
|  | FAS | [Hs00236330_m1](https://www.thermofisher.com/taqman-gene-expression/product/Hs00236330_m1?CID=&ICID=&subtype=) | 5’Fam3’NFQ-MGB |
|  | MAP3K5 | [Hs00178726_m1](https://www.thermofisher.com/taqman-gene-expression/product/Hs00178726_m1?CID=&ICID=&subtype=) | 5’Fam3’NFQ-MGB |
|  | TNBX | [Hs00372889_g1](https://www.thermofisher.com/taqman-gene-expression/product/Hs00372889_g1?CID=&ICID=&subtype=) | 5’Fam3’NFQ-MGB |
|  | IGF2BP3 | [Hs00559907_g1](https://www.thermofisher.com/taqman-gene-expression/product/Hs00559907_g1?CID=&ICID=&subtype=) | 5’Fam3’NFQ-MGB |
|  | LIN28b | [Hs01013729_m1](https://www.thermofisher.com/taqman-gene-expression/product/Hs01013729_m1?CID=&ICID=&subtype=) | 5’Fam3’NFQ-MGB |
|  | NETO2 | [Hs00983147_g1](https://www.thermofisher.com/taqman-gene-expression/product/Hs00983147_g1?CID=&ICID=&subtype=) | 5’Fam3’NFQ-MGB |
|  | CHD7 | [Hs00215010_m1](https://www.thermofisher.com/taqman-gene-expression/product/Hs00215010_m1?CID=&ICID=&subtype=) | 5’Fam3’NFQ-MGB |
|  | BCAT1 | [Hs00398962_m1](https://www.thermofisher.com/taqman-gene-expression/product/Hs00398962_m1?CID=&ICID=&subtype=) | 5’Fam3’NFQ-MGB |
|  | JAZF1F | AGCTCCATGTGCATGAATGG | - |
|  | JAZF1R | TTGAATGGTTTGCGGACACG | - |
|  | JUNDF | ACGAGCTCACAGTTCCTCTA | - |
|  | JUNDR | GCTGGTTCTGCTTGTGTAAATC | - |
|  | MYCF | TCCCTCCACTCGGAAGGAC |  |
|  | MYCR | CTGGTGCATTTTCGGTTGTTG |  |
|  | RPS18F | GGATGAGGTGGAACGTGT | - |
|  | RPS18R | CTAGGACCTGGCTGTATTTTC | - |
