# Supplementary material for: Comparison of gene expression profiles between human erythroid cells derived from fetal liver and adult peripheral blood
Source: PeerJ. 2018 Aug 31;6:e5527. doi: 10.7717/peerj.5527 (PMC6120446; doi:10.7717/peerj.5527)
Supplement: Table S6 [file peerj-06-5527-s011.docx]

| TF | NES^a^ | # targets (genes) | #motifs/tracks |
| --- | --- | --- | --- |
| FL up-regulated genes: | | | |
| MYC | 6.262 | 361 | 0/6 |
| MAX | 5.625 | 370 | 0/11 |
| MXI1 | 3.906 | 224 | 0/2 |
| FOXA1 | 3.651 | 99 | 4/0 |
| DBP | 3.590 | 48 | 2/0 |
| USP39 | 3.505 | 59 | 2/0 |
| MYB | 3.302 | 145 | 4/0 |
| PITX2 | 3.385 | 30 | 1/0 |
| ZBTB33 | 3.378 | 63 | 4/0 |
| NMRAL1 | 3.349 | 22 | 1/0 |
| GCM1 | 3.330 | 83 | 3/0 |
| ZNF407 | 3.283 | 19 | 0/1 |
| ZNF143 | 3.265 | 54 | 2/0 |
| CREB3L1 | 3.146 | 70 | 3/0 |
| SIN3A | 3.074 | 186 | 0/1 |
| POLR3A | 3.025 | 72 | 1/0 |
| AB up-regulated genes: | | | |
| GATA1 | 5.420 | 213 | 4/2 |
| RELA | 4.225 | 59 | 6/0 |
| PITX3 | 4.202 | 23 | 1/0 |
| CEBPA | 4.084 | 76 | 5/0 |
| SRF | 3.890 | 53 | 3/0 |
| CEBPD | 3.869 | 50 | 0/2 |
| IKZF2 | 3.768 | 59 | 4/0 |
| YY1 | 3.658 | 58 | 2/0 |
| PAX3 | 3.512 | 51 | 5/0 |
| STAT5A | 3.381 | 26 | 0/1 |
| TBX21 | 3.353 | 15 | 1/0 |
| POU2F2 | 3.232 | 19 | 0/1 |

1. NES (Normalized Enrichment Score): enrichment score of the motif or the maximal enrichment score for a given TF.
